# Early consequences of allopolyploidy alter floral evolution in *Nicotiana* (Solanaceae)

**DOI:** 10.1186/s12870-019-1771-5

**Published:** 2019-04-27

**Authors:** Elizabeth W. McCarthy, Jacob B. Landis, Amelda Kurti, Amber J. Lawhorn, Mark W. Chase, Sandra Knapp, Steven C. Le Comber, Andrew R. Leitch, Amy Litt

**Affiliations:** 1 Department of Botany and Plant Sciences, University of California, Riverside, Riverside, CA 92521 USA; 2 Department of Biology, University of Florida, Gainesville, FL 32611 USA; 3 Florida Museum of Natural History, University of Florida, Gainesville, FL 32611 USA; 4 Royal Botanic Gardens, Kew, Richmond, Surrey, TW9 3DS UK; 5 Department of Environment and Agriculture, Curtin University, Bentley, Western Australia 6102 Australia; 6 Natural History Museum, London, SW7 5BD UK; 7 School of Biological and Chemical Sciences, Queen Mary University of London, Mile End Road, London, E1 4NS UK; 8 Present address: Department of Biological Sciences, SUNY Cortland, Cortland, NY 13045 USA

**Keywords:** Ancestral character state reconstruction, Evolution, Flower color, Flower morphology, Geometric morphometrics, Hybridization, *Nicotiana*, Polyploidy

## Abstract

**Background:**

Polyploidy has played a major role in angiosperm evolution. Previous studies have examined polyploid phenotypes in comparison to their extant progenitors, but not in context of predicted progenitor phenotypes at allopolyploid origin. In addition, differences in the trends of polyploid versus diploid evolution have not been investigated. We use ancestral character-state reconstructions to estimate progenitor phenotype at allopolyploid origin to determine patterns of polyploid evolution leading to morphology of the extant species. We also compare trends in diploid versus allopolyploid evolution to determine if polyploidy modifies floral evolutionary patterns.

**Results:**

Predicting the ancestral phenotype of a nascent allopolyploid from reconstructions of diploid phenotypes at the time of polyploid formation generates different phenotype predictions than when extant diploid phenotypes are used, the outcome of which can alter conclusions about polyploid evolution; however, most analyses yield the same results. Using ancestral reconstructions of diploid floral phenotypes indicate that young polyploids evolve shorter, wider corolla tubes, but older polyploids and diploids do not show any detectable evolutionary trends. Lability of the traits examined (floral shape, corolla tube length, and corolla tube width) differs across young and older polyploids and diploids. Corolla length is more evolutionarily labile in older polyploids and diploids. Polyploids do not display unique suites of floral characters based on both morphological and color traits, but some suites of characters may be evolving together and seem to have arisen multiple times within *Nicotiana*, perhaps due to the influence of pollinators.

**Conclusions:**

Young polyploids display different trends in floral evolution (shorter, wider corolla tubes, which may result in more generalist pollination) than older polyploids and diploids, suggesting that patterns of divergence are impacted by the early consequences of allopolyploidy, perhaps arising from genomic shock and/or subsequent genome stabilization associated with diploidization. Convergent evolution in floral morphology and color in *Nicotiana* can be consistent with pollinator preferences, suggesting that pollinators may have shaped floral evolution in *Nicotiana*.

**Electronic supplementary material:**

The online version of this article (10.1186/s12870-019-1771-5) contains supplementary material, which is available to authorized users.

## Background

Polyploidy, or whole genome duplication, is a widespread phenomenon in angiosperms. All angiosperms have had at least one whole genome duplication in their evolutionary history [1], ~ 15% of speciation events in angiosperms and ~ 31% in ferns involve polyploidy [2], and 24% of extant vascular plants are neopolyploid [3]. Polyploidy may increase adaptability to new environments [4], but newly established polyploids are rare and therefore are at a disadvantage because they are much more likely to receive pollen from diploids, which may be incompatible due to the difference in ploidy [5], or may self-fertilize, leading to inbreeding depression. Many crop species, such as wheat, oilseed rape, coffee, and cotton, are allopolyploid, involving both whole genome duplication and interspecific hybridization [6].

The merger of two distinct genomes in one allopolyploid nucleus may result in ‘genomic shock’ [7], which yields changes in gene expression [8–12], chromosomal rearrangements [13, 14], increase of transposable element activity [15, 16], alterations of physiological processes [17, 18], changes in morphology [19, 20], and niche shifts [21]. These processes and their results can isolate newly formed allopolyploids from their diploid progenitors and may facilitate their establishment as a new species. The new combinations of traits that can result from genomic shock associated with allopolyploidy may allow them to respond differently than diploids to evolutionary pressures. In long-term evolution experiments with yeast grown on poor carbon-source media, tetraploid yeast adapted to the medium more rapidly than either haploid or diploid yeasts [22, 23]. Tetraploid yeast also accumulated a greater diversity of adaptive mutations, suggesting that tetraploids may have evolutionary potential that diploid and haploid yeasts lack [23]. However, diploid yeast consistently displays higher growth fitness than haploid, triploid, and tetraploid yeasts in multiple environmental contexts in short-term growth experiments [24]. Although growth in yeast and the evolution of complex traits in angiosperms may be on a different scale, they are both controlled by regulatory networks and biochemical pathways. Therefore, these yeast results suggest that tetraploids may be at a fitness disadvantage in the short-term, but may be more adaptable in the long-term, especially in harsh and stressful conditions [22–25]. The ability of polyploids to adapt to harsh environments has been proposed as one hypothesis for the persistence and increased diversification of polyploids after major ecological events such as the mass extinction event at the Cretaceous-Paleogene boundary [25–27].

Previous studies in angiosperms have investigated allopolyploid phenotypic evolution with respect to plant biomass [19, 28], photosynthetic capacity [29], non-photochemical quenching [18], defense response to herbivory [17], and flower morphology and color [20, 30–32]. These studies have compared allopolyploid phenotypes to those of their diploid progenitors to evaluate whether allopolyploids display novel traits or combinations of traits, but these studies have not addressed whether allopolyploids follow different evolutionary trends than diploids. In addition, these studies do not take into account the fact that the diploid progenitor species have also been evolving since allopolyploid origin. Therefore, the phenotypes of the progenitors at the time of allopolyploid origin may have been different from those of the extant species that are used to evaluate allopolyploid phenotypes and evolution. This divergence of phenotypes is particularly likely to be true in older allopolyploid species. Using extant progenitors may, thus, introduce error into our interpretation of how allopolyploids have evolved. Previously, studies have reported differences in the short- and long-term consequences of allopolyploidy for genome structure [13, 15, 16, 33, 34], but less is known about short- and long-term consequences of allopolyploidy on phenotype, and none, as far as we are aware, has used ancestral character-state reconstruction to predict the phenotype of the progenitor diploids at the time of polyploid origin. In this study, we use ancestral character state reconstructions to compare the evolutionary responses of allopolyploids and diploids and to determine whether using reconstructed progenitor phenotypes modifies our conclusions about polyploid evolution.

*Nicotiana* consists of 76 species, about half of which arose from six independent allotetraploid events at different time points (ca. 0.4, 0.6, 0.7, 1.4, 4.3, and 6 million years ago; Table 1; [35]. In addition, synthetic allopolyploids that were created in the lab ( [19]; K.Y. Lim, Queen Mary, University of London) from the same progenitor species as natural allopolyploids are available. *Nicotiana* has been well studied phylogenetically [36–40], and putative parentage of all allopolyploid species/groups has been determined. *Nicotiana* displays considerable diversity in floral morphology and color [20, 31, 41], facilitating study of the effects of allopolyploidy on floral evolution. *Nicotiana* allopolyploids can display transgressive morphologies that fall outside the range of their diploid progenitors and are thought to have evolved shorter, wider corolla tubes than expected, assuming the nascent polyploid has a morphology predicted by the morphologies of the flowers of extant diploids [20].

**Table 1:** *Nicotiana* allotetraploids (except section *Suaveolentes*) and their diploid progenitors and ages [35]

Polyploid	Section	Maternal Progenitor	Paternal Progenitor	Approximate Age (myo)
*N. tabacum*	*Nicotiana*	*N. sylvestris*	*N. tomentosiformis*	0.6
*N. rustica*	*Rusticae*	*N. paniculata*	*N. undulata*	0.7
*N. arentsii*	*Undulatae*	*N. undulata*	*N. wigandioides*	0.4
*N. clevelandii*	*Polydicliae*	*N. obtusifolia*	*N. attenuata*	1.4
*N. quadrivalvis*	*Polydicliae*	*N. obtusifolia*	*N. attenuata*	1.4
*N. nesophila*	*Repandae*	*N. sylvestris*	*N. obtusifolia*	4.3
*N. nudicaulis*	*Repandae*	*N. sylvestris*	*N. obtusifolia*	4.3
*N. repanda*	*Repandae*	*N. sylvestris*	*N. obtusifolia*	4.3
*N. stocktonii*	*Repandae*	*N. sylvestris*	*N. obtusifolia*	4.3

In this study, we compare floral evolution in allopolyploids to that observed in diploids to address the following questions. 1) Do allopolyploids have novel suites of floral characters not found in diploids? 2) Do allopolyploids display different evolutionary trends in floral morphology than diploids? 3) Are there differences between phenotypic evolution immediately following allopolyploidy versus that observed over longer time scales? 4) Do reconstructed progenitor phenotypes alter interpretation of polyploid evolution compared with predictions using values from extant diploids directly?

## Results

### Concatenated dataset yielded well-supported tree

**Fig. 1:**
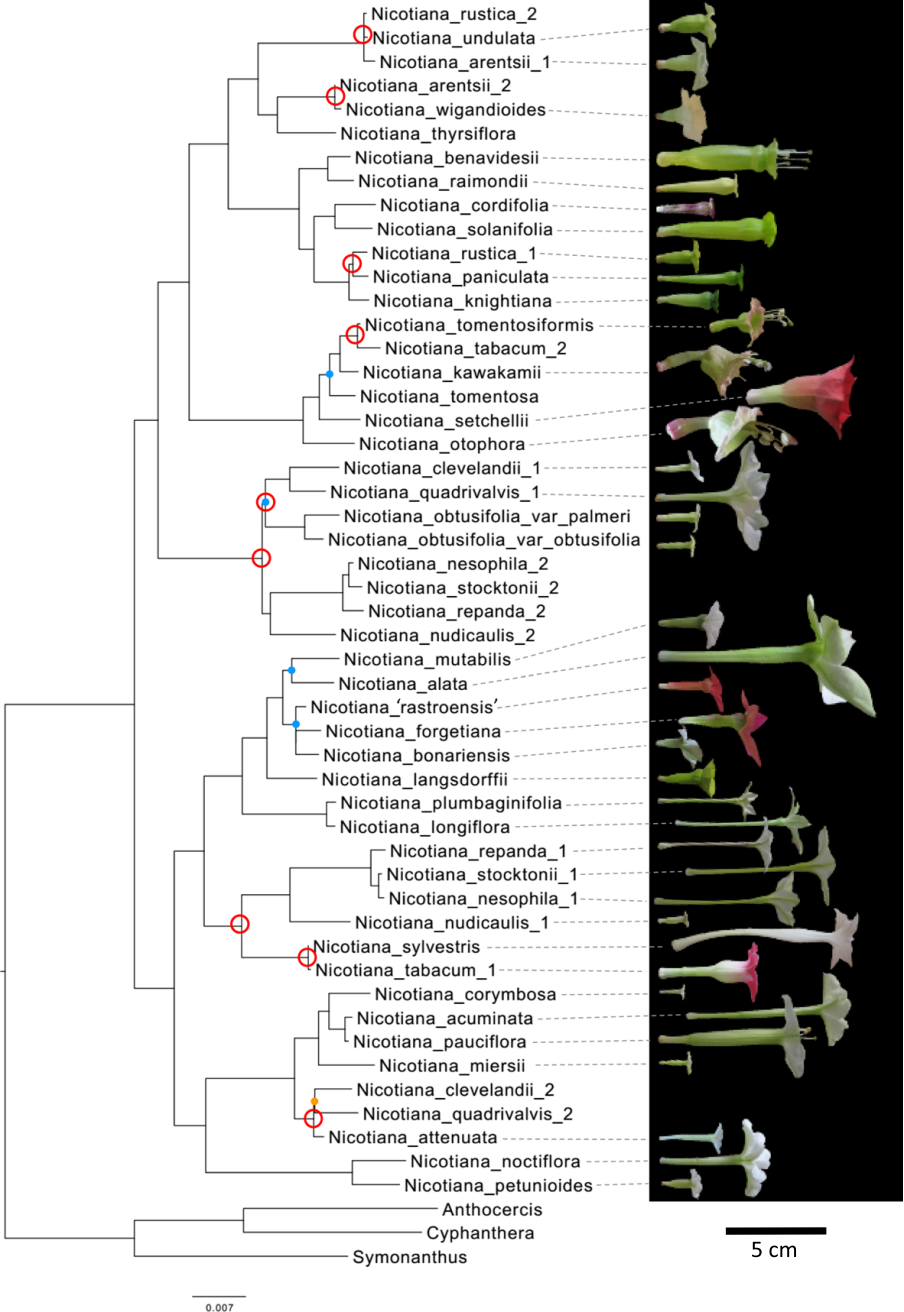
Phylogenetic tree of diploid and allopolyploid *Nicotiana* species. Tree reconstructed from the concatenated dataset based on maximum likelihood (ML) and Bayesian analyses is well-supported at almost all nodes. Plain nodes: ML bootstrap > 70%, posterior probability > 0.95; node with orange dot: ML bootstrap > 70%, posterior probability < 0.95; nodes with blue dot: ML bootstrap < 70%, posterior probability < 0.95; nodes with red circles: nodes of polyploid origin for estimating reconstructed progenitor phenotypes. Side flower photographs to scale, bar = 5 cm

In order to determine whether allopolyploids and diploids display different evolutionary trends, we reconstructed ancestral character states, which requires a well-supported phylogenetic tree representing species relationships and detailed character states for extant species. Previous phylogenetic studies in *Nicotiana* [36–39] elucidated species relationships and hybrid origins with strong support, but often lacked support for backbone nodes because they were based on single DNA marker sequences. Our concatenated dataset, which uses sequences obtained from these previous studies with additional sequences generated in this study (Additional file 1: Table S1), produced a well-supported tree with > 70% bootstrap support from maximum likelihood (ML) analyses for all except five nodes and > 0.95 posterior probabilities from Bayesian analyses for all except four nodes. In these analyses the backbone is also well supported (Fig. 1).

In our tree, the positions of allopolyploid sections, and therefore the inferred diploid progenitors, are congruent with those found in previous studies [36–38]. In addition, our results suggest that diploid *Nicotiana* can be separated into two large clades, consisting of 1) sections *Undulatae*, *Paniculatae*, *Tomentosae*, and *Trigonophyllae* and 2) sections *Alatae*, *Sylvestres*, *Petunioides*, and *Noctiflorae*. Sister relationships were observed for sections *Alatae* and *Sylvestres*, *Petunioides* and *Noctiflorae*, and *Undulatae* and *Paniculatae*, whereas section *Tomentosae* was sister to sections *Undulatae* and *Paniculatae*, and section *Trigonophyllae* was sister to sections *Undulatae*, *Paniculatae*, and *Tomentosae*.

### Floral variation in Nicotiana

Geometric morphometric analysis of floral shape in *Nicotiana* allopolyploids and diploids yielded a similar morphospace based on principal components 1 and 2 to that obtained previously [20]. The morphospace consists of two diagonal axes: round to stellate floral limb outline, and relatively small to relatively large floral tube opening (‘relative’ because all shapes are scaled to the same size in this analysis; Additional file 2: Figure S1). Principal component 1 (PC1) accounts for 58.84% of the variation in the dataset and PC2 for 19.41% of the variation. Across the corolla size dataset, corolla tube length ranged from 0.84 to 9.36 cm and tube width ranged from 0.14 to 1.65 cm (Additional file 2: Figure S1) based on floral averages calculated from measurements of five replicate photographs from each flower.

### Floral evolution in diploids versus allopolyploids

**Fig. 2:**
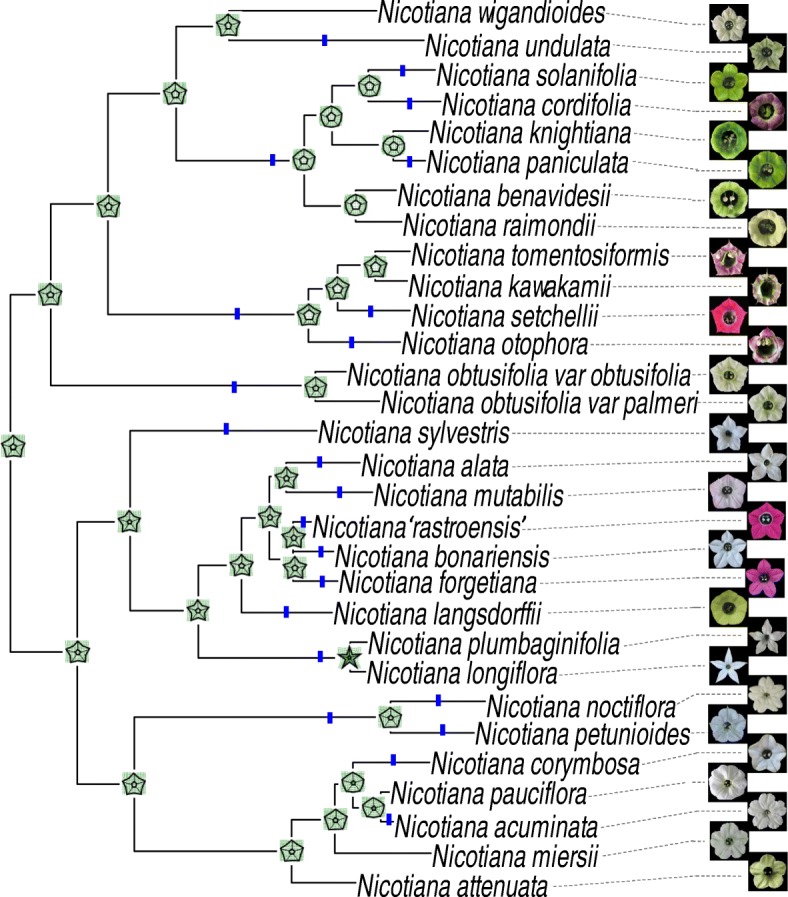
Ancestral character state reconstructions of floral limb shape on a diploid tree. Reconstructed values of floral limb shape represented by thin plate splines from the geometric morphometric morphospace (obtained using reconstructed (PC1, PC2) coordinates) at each internal node. Substantial shifts (greater than 10% of the range of shape variation) in floral limb shape marked on branches with blue lines. Front flower photos scaled to the same size to show only changes in shape

**Fig. 3:**
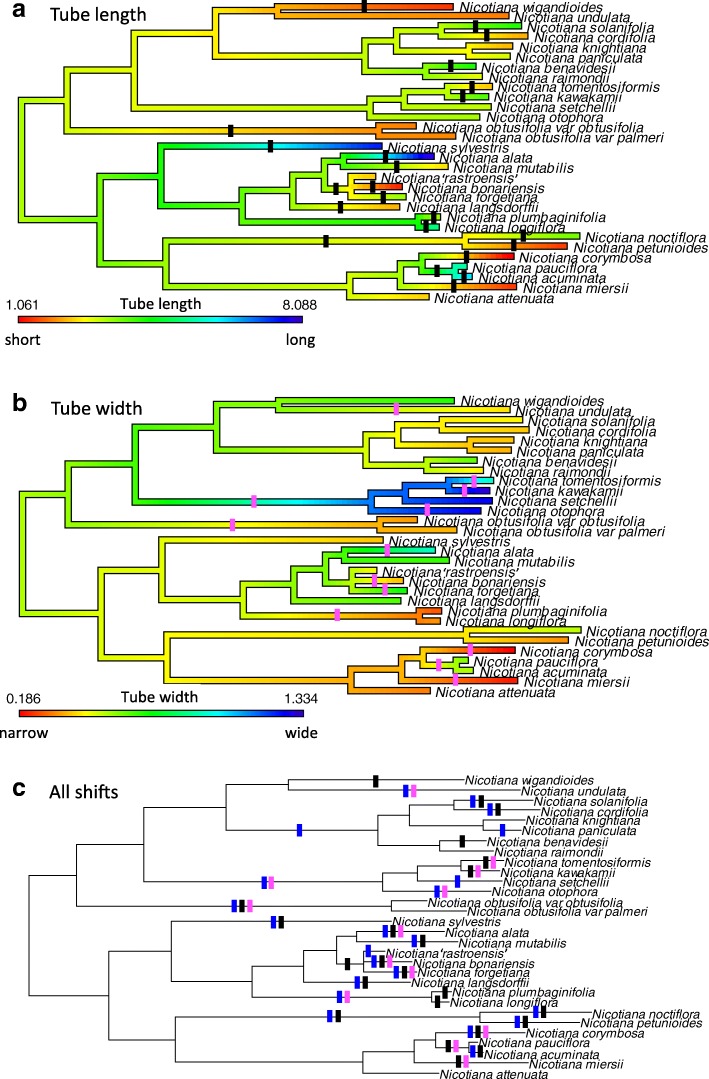
Ancestral character state reconstructions of corolla tube length and width on a diploid tree. Reconstructed values of corolla tube length (**a**) and width (**b**) represented as a heat map across the tree; red = short/narrow, blue = long/wide. Substantial shifts (greater than 10% of the range of tube length or width variation) marked on branches with black (length) or pink (width) lines. **c** Tree with all shifts in floral limb shape (blue), length (black), or width (pink) to determine on which branches shifts in multiple traits occur

To determine evolutionary trends of diploid morphology, we used ancestral character state reconstruction to predict substantial shifts in floral morphology across the diploid-only tree. A few examples of substantial shifts include: a shift to a more stellate floral outline on the branch leading to the most recent common ancestor of *N. plumbaginifolia* and *N. longiflora* (Fig. 2), a shift to a longer corolla tube on the branch leading to *N. sylvestris* (Fig. 3a), and a shift to a smaller corolla tube width on the branch leading to *N. miersii* (Fig. 3b). The number of shifts in the evolution of floral shape (22 shifts; Fig. 2) is similar to that seen in the evolution of tube length (23 shifts; Fig. 3a), whereas shifts in the evolution of tube width are less common (13 shifts; Fig. 3b). In addition, 71% of branches that have shifts have them in more than one trait (Fig. 3c), demonstrating that shifts in multiple traits tend to co-occur.

We tested whether each floral trait displayed phylogenetic signal, that is, whether closely related species tend to have similar morphology, using both Blomberg’s K and Pagel’s λ. All floral traits showed less phylogenetic signal than predicted by a Brownian motion model of trait evolution (K < 1, Table 2), but only the results for tube length failed to reject the null hypothesis of no phylogenetic signal. We obtained similar results for Pagel’s λ; tube width and both PC1 and PC2 were significantly different from λ = 0 (no phylogenetic signal), but tube length was not (Table 2). These results suggest that the evolution of tube length is less constrained by phylogeny than that of the other floral traits.

**Table 2:** Phylogenetic signal tests

Floral trait	Blomberg’s K	Randomization test (H_0_: no signal)	Pagel’s λ	H_0_: no signal
PC1	0.549	*p* = 0.002	0.857	*p* = 0.00013
PC2	0.786	*p* = 0.001	0.99995	*p* = 3.4 × 10^−5^
Tube length	0.275	*p* = 0.213	6.61 × 10^−5^	*p* = 1
Tube width	0.790	p = 0.002	0.918	*p* = 1.68 × 10^−5^

**Fig. 4:**
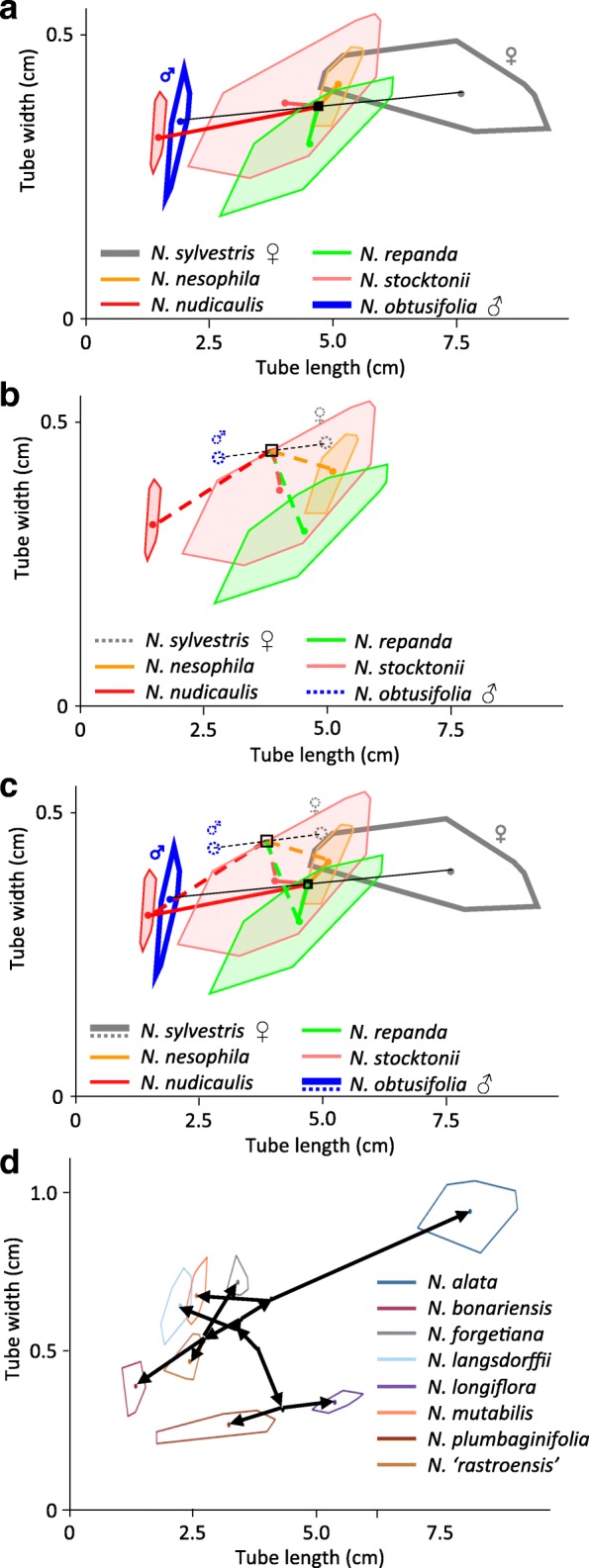
Reconstructed phenotypes in the context of polyploid and diploid evolution. Convex polygons enclose the space taken up by all flower averages and the colored point represents the species mean for each species/accession. **a**-**c** Allopolyploid section *Repandae* in tube length and width in the context of extant progenitor phenotypes (**a**), reconstructed progenitor phenotypes (**b**), and both (**c**). Allopolypoids have filled polygons; diploid progenitors have outlined polygons and are labelled with ♀ for maternal and ♂ for paternal. The progenitor midpoint is denoted with a black square. Reconstructed progenitors are marked with dotted circles and the reconstructed progenitor midpoint is a square with a dotted black outline. Colored lines connect progenitor midpoints with allopolyploid means (solid = extant; dashed = reconstructed). **d** Diploid section *Alatae* in tube length and width. Black dots represent reconstructed phenotypes at internal nodes on the phylogenetic tree. Arrows denote direction of evolution based on phylogenetic relationships

**Fig. 5:**
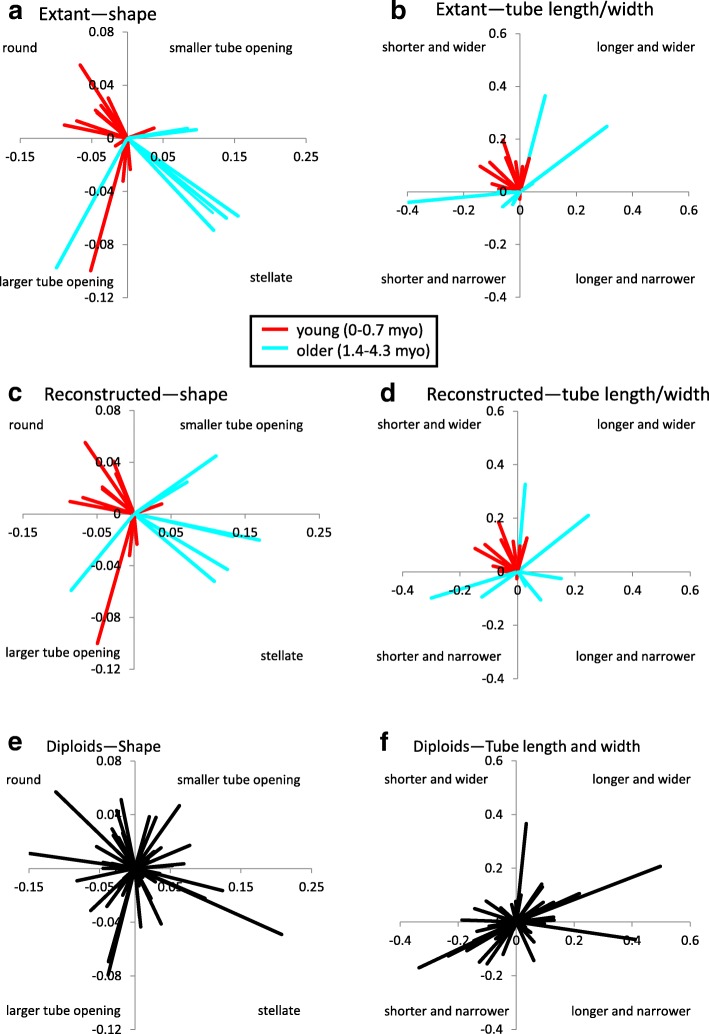
Trends in allopolyploid versus diploid floral divergence. Trends in evolution for extant allopolyploid floral limb shape (**a**), extant allopolyploid tube length and width (**b**), reconstructed allopolyploid floral limb shape (**c**), reconstructed allopolyploid tube length and width (**d**), diploid floral limb shape (**e**), and diploid tube length and width (**f**). Lines represent vector from the progenitor midpoint to the allopolyploid mean in (**a**-**d**) and the origin represents the progenitor midpoint. Young allopolyploids (0–0.7 myo) shown in red; older allopolyploids (1.4–4.3 myo) shown in light blue. In (**e**-**f**), lines represent the difference in reconstructed values between successive nodes on the tree and the origin represents the older node. Labels in the quadrants denote the phenotype toward which the vectors in that quadrant are evolving

To estimate the progression of floral morphological evolution in diploids, we quantified the direction and magnitude of changes between successive internal nodes and between extant taxa and their reconstructed most recent ancestor on the diploid tree. The changes seen between reconstructed internal nodes are represented by the arrows in Fig. 4. We compared these with the direction and magnitude of the morphological change in allopolyploids as measured by the distance between the progenitor midpoint (the average of the means of each progenitor species) and the mean of each allopolyploid species/accession, following the methods of McCarthy et al. [20]. We then compared trends in floral morphological evolution between diploids and allopolyploids (Fig. 5).

Based on the graphs in Fig. 5, diploids do not display any clear trends in evolution because the estimated progression of evolution is not significantly different from a uniform circular distribution around the origin in either floral limb shape (Moore-Rayleigh, R* = 0.0237, *N* = 58, *p* < 0.999, significance threshold of α = 0.05 is 0.0036 after Bonferroni correction) or in tube length and width (Moore-Rayleigh, R* = 0.365, *N* = 58, *p* < 0.90; Fig. 5e, f; Table 3). Similarly, overall trends in the direction of evolution in floral limb shape in allopolyploids are not significantly different from a uniform circular distribution when the complete polyploid dataset is analyzed (Moore-Rayleigh, R* = 0.433, *N* = 20, *p* < 0.90) or when young (0–0.7 million years old (myo); Moore-Rayleigh, R* = 1.27, *N* = 13, *p* < 0.01, significance threshold of α = 0.05 is 0.0036 after Bonferroni correction) and older (1.4–4.3 myo; Moore-Rayleigh, R* = 1.202, *N* = 7, *p* < 0.025, significance threshold of α = 0.05 is 0.0036 after Bonferroni correction) allopolyploids are analyzed separately (Fig. 5a, c; Table 3). For the complete polyploid dataset, patterns of evolution in corolla length and width are significantly different from a uniform circular distribution (Moore-Rayleigh, R* = 1.57, *N* = 20, *p* < 0.001; Fig. 5b, d; Table 3), suggesting that polyploids tend to evolve shorter, wider corolla tubes as also concluded in McCarthy et al. [20]. For the young polyploids, patterns of evolution are again skewed toward shorter and wider corolla tubes (Moore-Rayleigh, R* = 1.65, *N* = 13, *p* < 0.001); however, older polyploids are not significantly different from a uniform circular distribution (Moore-Rayleigh, R* = 0.437, *N* = 7, *p* < 0.90; Fig. 5b, d; Table 3). These results suggest that polyploids have diverged along a more similar path than diploids, especially early in allopolyploid evolution.

**Table 3:** Moore-Rayleigh test results (** denotes significance: ɑ = 0.05 after Bonferroni correction is 0.0036)

Trait	Group	Extant/Reconstructed	N	R*	*P*-value
Shape	All diploids	–	58	0.0237	p < 0.999
Shape	All polyploids	Extant	20	0.433	p < 0.90
Shape	Young polyploids	Extant	13	1.27	p < 0.01
Shape	Old polyploids	Extant	7	1.20	p < 0.025
Shape	All polyploids	Reconstructed	20	0.314	p < 0.90
Shape	Young polyploids	Reconstructed	13	1.19	p < 0.025
Shape	Old polyploids	Reconstructed	7	1.28	p < 0.01
Length/Width	All diploids	–	58	0.365	p < 0.90
Length/Width	All polyploids	Extant	20	1.57	p < 0.001**
Length/Width	Young polyploids	Extant	13	1.65	p < 0.001**
Length/Width	Old polyploids	Extant	7	0.437	p < 0.90
Length/Width	All polyploids	Reconstructed	20	1.01	p < 0.10
Length/Width	Young polyploids	Reconstructed	13	1.64	p < 0.001**
Length/Width	Old polyploids	Reconstructed	7	0.234	p < 0.90

### Reconstructed progenitor phenotypes do not alter interpretation of allopolyploid evolution in Nicotiana

**Fig. 6:**
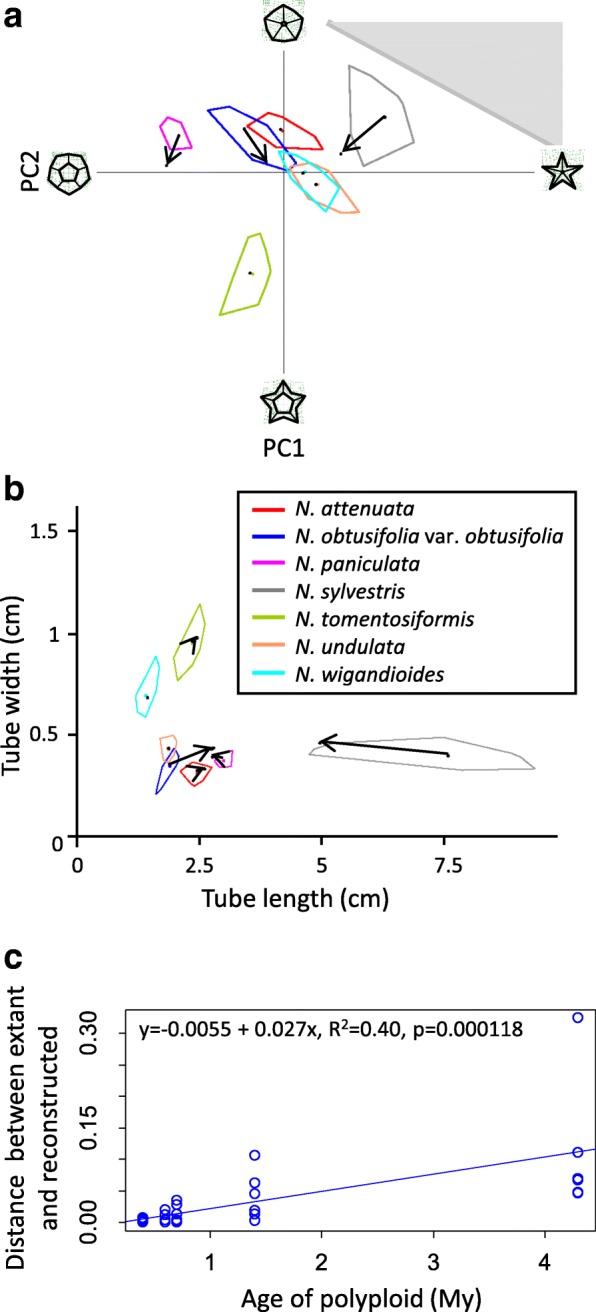
Differences between extant and reconstructed progenitor phenotypes in floral limb shape (**a**) and tube length and width (**b**). The legend for the species depicted in (**a**) and (**b**) is shown in (**b**). Each extant species is represented by a convex polygon: polygon encloses the space taken up by all flower averages and the colored point represents the species mean. Reconstructed phenotypes are delineated by black points. Arrows connect the extant species mean to the reconstructed phenotype for each diploid progenitor. In (**a**), gray triangle represents impossible shapes where the landmarks that denote the floral tube opening cross each other, creating negative space. Thin plate splines show the extent of shape variation in the morphospace. **c** Distance between extant progenitor mean and reconstructed phenotype in shape and corolla tube dimensions plotted versus allopolyploid age

We hypothesized that the difference between reconstructed and extant progenitor morphology would increase with polyploid age (polyploid parentage and age are found in Table 1). To test this, we measured the distance between extant and reconstructed progenitor phenotypes for *Nicotiana* polyploids of different ages in our floral trait morphospaces. About half (8 of 14) of the reconstructed phenotypes showed differences from their extant counterparts (Fig. 6a, b). As predicted, the distance between extant and reconstructed diploid progenitor phenotypes increased with allopolyploid age (Fig. 6c).

To determine whether the use of reconstructed phenotypes alters the interpretation of allopolyploid divergence, we compared the results of our analyses of allopolyploid evolution using reconstructed versus extant progenitor phenotypes. Extant and reconstructed progenitor midpoints differed in 60% of cases (6 of 10) based on floral limb shape and corolla tube length and width data (Fig. 4a, b, c; Additional file 3: Figure S2; Additional file 4: Figure S3), and these changes in the progenitor midpoint resulted in differences in the direction of allopolyploid divergence in 7 and 27% of allopolyploids in floral limb shape and tube length and width, respectively (Fig. 5a-d). For example, *N. nesophila*, *N. repanda*, and *N. stocktonii*, which have the same two progenitors, have corollas that are longer and wider, shorter and narrower, and shorter and wider, respectively, compared to their extant progenitor midpoint (Fig. 4a, c). When compared to their reconstructed progenitor midpoint, however, they are all longer and narrower (Fig. 4b, c).

Comparison of the results of Moore-Rayleigh tests based on either extant or reconstructed progenitor phenotypes show that reconstructed progenitor phenotypes do not change the response of allopolyploids in floral evolution in most of these analyses. For floral limb shape, trends in the direction of evolution using reconstructed progenitor phenotypes are not significantly different from a uniform circular distribution when the complete polyploid dataset is analyzed (Moore-Rayleigh, R* = 0.314, *N* = 20, *p* < 0.90) or when young (Moore-Rayleigh, R* = 1.19, *N* = 13, *p* < 0.025, significance threshold of α = 0.05 is 0.0036 after Bonferroni correction) and older (Moore-Rayleigh, R* = 1.28, *N* = 7, *p* < 0.01, significance threshold of α = 0.05 is 0.0036 after Bonferroni correction) polyploids are analyzed separately (Fig. 5a, c; Table 3). For corolla tube length and width, patterns of evolution are skewed toward shorter and wider corolla tubes for young polyploids (Moore-Rayleigh, R* = 1.64, *N* = 13, *p* < 0.001), but are not significantly different from a uniform circular distribution for older polyploids (Moore-Rayleigh, R* = 0.234, *N* = 7, p < 0.90) as observed for extant phenotypes (Fig. 5b, d; Table 3). In contrast, corolla tube length and width evolution is not significantly different from a uniform circular distribution when the complete polyploid dataset is analyzed based on reconstructed progenitor phenotypes (Moore-Rayleigh, R* = 1.01, *N* = 20, *p* < 0.10), whereas it is skewed towards shorter and wider tubes when extant progenitor phenotypes are used (Fig. 5b, d; Table 3).

### Allopolyploids sometimes display suites of floral characters not observed in diploids

To determine whether allopolyploids display suites of floral characters that are not found in diploids, we identified evolutionary shifts in floral characters and determined whether any of these shifts represent convergent evolution. When only morphological characters were used, the two *N. quadrivalvis* accessions and the four *N. tabacum* accessions were placed in the same convergent regime with the following characters: a stellate floral shape with a relatively large tube opening compared to floral limb breadth, medium tube length (average from 2.90–4.67 cm), and large tube width (average from 0.67–0.94 cm; Additional file 5: Figure S4). Both *N. quadrivalvis* copies were included in this convergent regime, but it included only the maternal copy of the *N. tabacum* accessions; the paternal copy of *N. tabacum* grouped with its paternal progenitor and related diploids. Because each allopolyploid copy has a different phylogenetic context due to the different evolutionary histories of the progenitors that contributed each copy, the surface program can place the two copies in different regimes even though the same morphology is entered for both copies. Nevertheless, these results suggest that *N. quadrivalvis* and *N. tabacum* allopolyploids possess a suite of floral characters distinct from those found in *Nicotiana* diploids. In addition, both copies of the related allopolyploids *N. nesophila*, *N. repanda*, and *N. stocktonii* were grouped in a convergent regime with the following characters: a stellate floral shape with a relatively small tube opening compared to floral limb breadth and a long (average from 4.28–5.10 cm) and narrow (average from 0.31–0.42 cm) corolla tube (Additional file 5: Figure S4). Although this is unsurprising since the same morphology was input for both copies for each species, it suggests that these allopolyploid species display a suite of floral characters that is not shared with any diploid species, based on morphological data. Other allopolyploids were grouped with either their maternal progenitor, paternal progenitor, or both (Additional file 5: Figure S4).

**Fig. 7:**
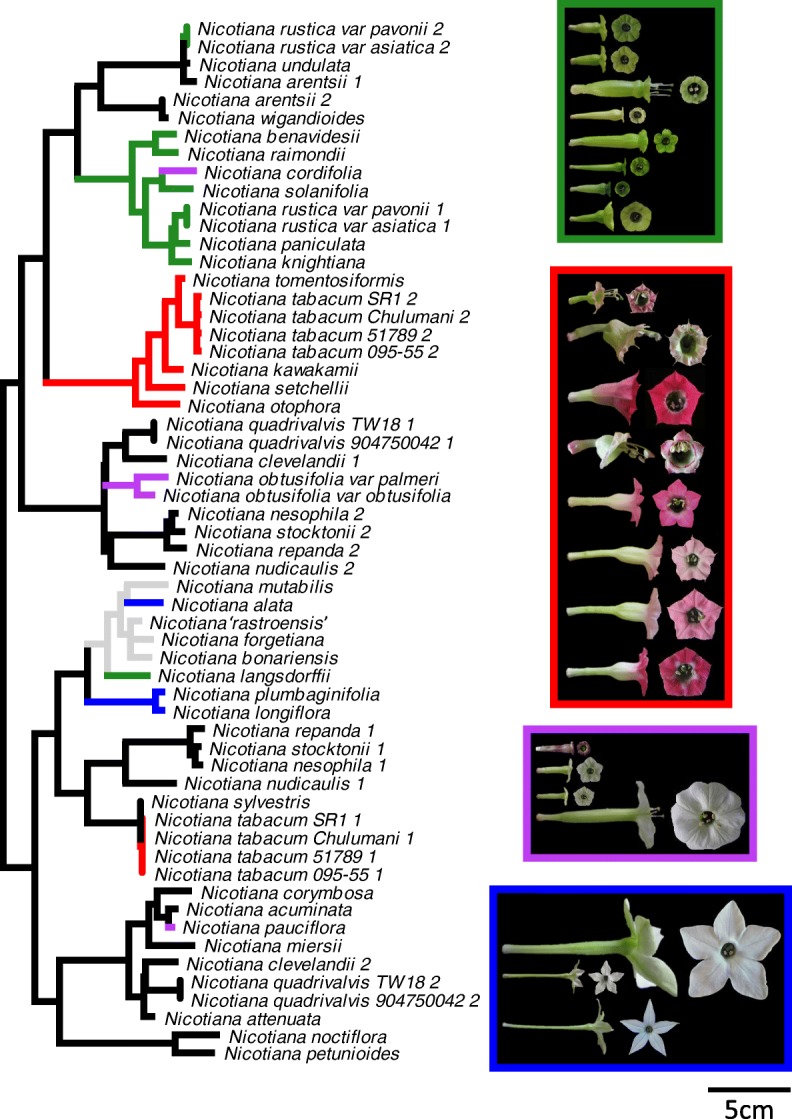
Convergent evolution analyses based on morphological and color traits. Morphological traits used include shape PC1, shape PC2, tube length, and tube width. Color traits used include the first three PCs of the spectral PCA. Shifts in color across the branches of the tree represent shifts in floral traits. Brightly colored branches denote suites of similar floral characters that have arisen independently in the evolution of *Nicotiana*; each color represents a different convergent regime. Green = the ‘green’ regime, red = the ‘pink’ regime, purple = the ‘high UV’ regime, and blue = the ‘white, stellate’ regime. The colors outlining the boxes with flower photographs correspond to these colors. Gray branches on the tree represent shifts in floral traits that were not placed into a convergent regime (they have a single origin). Flower photographs are to scale; scale bar = 5 cm

In the analyses with only color characters and with both morphological and color characters, all allopolyploids are grouped with either their maternal, paternal, or both progenitors (Fig. 7; Additional file 6: Figure S5). However, these analyses indicate that several convergent regimes are present within *Nicotiana*. It should be noted that the floral color PCA was performed with spectra that were normalized to the same area under the curve in order to group spectra with the same shape, and thus most likely similar pigments, instead of focusing on the brightness or concentration of pigment. Therefore, convergent regimes may include species with varying floral color saturation, i.e. some light flowers and some dark flowers, but should reflect differences in floral hue. In the floral color only analysis, the four convergent regimes identified correspond to green-, magenta/purple-, pink-, and UV-reflecting white-flowered species (Additional file 6: Figure S5), suggesting that these floral colors arose multiple times in *Nicotiana*.

In the morphology and color analysis, convergent regimes are similar to those obtained with the color only analysis, but with a few differences, suggesting that flowers with the same colors tend to have similar morphology. The ‘green’ regime also includes *N. knightiana* and groups species with flowers that are green, have a round shape with a relatively large opening compared to floral limb breadth, medium width (average from 0.38–0.65 cm), and some variation in length (average from 1.64–4.24 cm; Fig. 7). The ‘UV-reflecting white’ regime no longer includes *N. nudicaulis*, but does include *N. cordifolia* and is marked by species with flowers that have high UV to visual spectral ratio (*N. cordifolia* is purple, not UV-reflecting white), mostly round floral shape, medium length (average from 1.89–2.20 cm, except for *N. pauciflora*: 5.51 cm), and medium width (average from 0.35–0.60 cm; Fig. 7). The species composition of the ‘pink’ regime is identical to that recovered using only floral color data and comprises of flowers that are pink, magenta, or red (to humans) with stellate shape and relatively large tube opening compared to floral limb breadth, wide tubes (average from 0.81–1.33 cm), and some variation in tube length (average from 2.36–4.56 cm; Fig. 7). The fourth convergent regime, the ‘white, stellate’ regime, was not found in the color-only analyses and contains species with white stellate flowers, relatively small tube opening compared to floral limb breadth, and long corolla tubes (average 3.21–8.09 cm; Fig. 7). The presence of several convergent regimes across *Nicotiana* suggests that suites of floral characters may be evolving together, perhaps due to the influence of pollinators. In addition to convergent regimes, the surface analyses detected shifts in floral traits that were not associated with convergent evolution. Both these unique shifts and the convergent regimes tend to correspond to shifts in at least two traits identified using the ancestral reconstruction analyses (Figs. 3c and 7).

## Discussion

### Corolla length evolution is less labile in allopolyploids than diploids and may play a role in speciation

Evolutionary differences among young polyploids, older polyploids, and diploids are apparent in the relative lability of different floral traits. In polyploids, we measure lability as being less likely to overlap with the expected phenotype, represented by the mean of the progenitors (our null hypothesis). Polyploid groups with a lower percentage of accessions overlapping with the expected phenotype were considered more labile. In diploids, we measure lability by testing whether a trait displays phylogenetic signal; traits with no phylogenetic signal are more labile than traits that show phylogenetic signal. Because testing phylogenetic signal requires the input of a phylogenetic tree, we cannot use this metric to analyze polyploids. Although we cannot use the same metric to measure lability in both polyploids and diploids, we can rank the lability of the three floral traits (most labile to least labile) and compare this ranking order across young polyploids, older polyploids, and diploids to determine whether the same or different traits are relatively more labile in the three groups.

Our previous analyses suggest that corolla tube length may be less labile than floral limb shape or tube width in *Nicotiana* polyploids [20], which show less overlap with the expected phenotype. However, when we separate young and older polyploids and re-examine these data, we see a different pattern. In young polyploids, we observe greater overlap with the expected phenotype for tube length than floral limb shape, and least overlap for tube width [20]. Thus, tube width is the most labile trait in young polyploids, followed by floral limb shape, with tube length as least labile trait. In contrast, older polyploids are most labile in floral limb shape, followed by tube length, with tube width the least labile [20]. In diploids, corolla tube length is the most labile (no phylogenetic signal), whereas corolla tube width and floral limb shape are less labile (significant phylogenetic signal; Table 2). Thus, floral traits differ in lability across groups, suggesting that they may be under different evolutionary pressures.

Differences in the relative lability of corolla tube length may be linked to pollination. Length of corolla tubes is an important factor in the fit between flower and pollinator [42–44], and as a result, shifts in corolla tube or nectar spur length may facilitate reproductive isolation between species [45–47]. Consistent with this hypothesis, six of the 11 sister species pairs in our diploid tree show opposing shifts in corolla tube length: one towards a longer tube and the other towards a shorter tube (Fig. 3a). These 12 shifts comprise over half of the shifts in corolla tube length observed. These results, along with evidence that tube length evolution is not constrained by phylogeny, suggest that shifts in corolla tube length may play a role in species divergence, perhaps via pollinator-mediated selection. Pollinator relationships have only been elucidated for a subset of *Nicotiana* species; however, several of the sister species pairs that display shifts in tube length belong to section *Alatae*, the best studied section of *Nicotiana* in terms of pollination. These three pairs of sister species not only show shifts in tube length, but also shifts in their primary pollinators. Between sister species *N. alata* and *N. mutabilis*, longer-tubed *N. alata* is pollinated by hawkmoths [48], and shorter-tubed *N. mutabilis* is pollinated by hummingbirds [49]; hawkmoth-mediated selection has been shown to drive evolution of longer corolla tubes [45]. Shorter-tubed *N. bonariensis* is visited by small moths with tongues of similar length to its corolla tube, whereas its sister species, longer-tubed *N. forgetiana*, is primarily pollinated by hummingbirds [50]. Longer-tubed *N. longiflora* is pollinated by hawkmoths, but its shorter-tubed sister species, *N. plumbaginifolia*, is self-pollinating [50], consistent with the idea that outcrossing species are more likely to be subject to constant selective pressure from pollinators [50, 51] and therefore to maintain a longer corolla tube. These results demonstrate that shifts in corolla tube length can be correlated with specialization toward different pollinator types between species, suggesting that pollinators may influence the evolution of corolla tube length in *Nicotiana* and that this in turn may play a role in species divergence.

Similarly, older allopolyploid species that share the same origin also display divergence in corolla tube length [20]; Fig. 1). Within section *Polydicliae* (~ 1.4 million years old; [35], *N. clevelandii* has short corolla tubes, whereas those of *N. quadrivalvis* are much longer (Additional file 4: Figure S3). In section *Repandae* (~ 4.3 million years old; [35], *N. nudicaulis* has a short corolla tube, but its sister clade composed of *N. repanda*, *N. nesophila*, and *N. stocktonii* have much longer tubes (Fig. 4). The prevalence of corolla tube divergence in older polyploids is consistent with the divergence seen in diploid *Nicotiana* species. One aspect that distinguishes older polyploids and diploids from young polyploids is that they have undergone species diversification. Our current phylogenetic results support a single polyploid event from each diploid progenitor pair; older polyploid taxa have speciated since these single polyploid events and comprise sections including two or more species, whereas young polyploids have not undergone additional speciation and consist of only a single species per event. It is possible that divergence in tube length accompanies or facilitates speciation, perhaps via specialization for different pollinator types.

### Reconstructed progenitor phenotypes do not alter interpretation of allopolyploid evolution

Several studies have used ancestral state reconstruction of chromosome number to infer polyploidy events [52–55]. However, we are unaware of any study that has reconstructed progenitor phenotypes at the point of allopolyploid origin specifically to examine whether use of reconstructed versus extant progenitor phenotypes alters interpretation of morphological evolution of allopolyploids. Because extant diploid phenotypes may have diverged substantially from the phenotypes of the progenitors at the time of allopolyploid origin, using extant diploids to analyze trends in allopolyploid evolution may lead to inaccurate interpretations. Here, we show that reconstructed ancestral states of diploid progenitors at the point of allopolyploid origin differ from extant morphology about half of the time and nearly always for older allopolyploids as is expected with increased time for divergence (Fig. 6). These differences in progenitor phenotypes will also result in differences in the progenitor midpoint, which predicts allopolyploid morphology at its origin. Differences between the progenitor midpoint based on extant versus reconstructed progenitor phenotypes were observed in 60% of cases based on our character data (Fig. 4c; Additional file 3: Figure S2; Additional file 4: Figure S3).

However, in most analyses of allopolyploid evolution performed here, the conclusions are the same whether reconstructed or extant progenitor phenotypes are used. The exception is that there is no trend in the direction of evolution of corolla tube length and width of all polyploids when reconstructed progenitor phenotypes are used as opposed to a trend toward shorter and wider tubes when extant progenitor phenotypes are used (Fig. 5b, d). Because the results for young and older polyploids are consistent between analyses, the difference in the response of all polyploids between analyses suggests that it is the immediate and short-term consequences of polyploidy that result in the evolution of shorter and wider tubes, instead of differences between polyploids and diploids per se.

### Convergent evolution in Nicotiana

Our results show that *Nicotiana* allopolyploids do not have suites of floral characters distinct from diploid species when both morphological and color traits are considered and only rarely display distinct suites of morphological characters. However, our analyses recovered convergent ‘regimes’ that seem to have evolved multiple times independently within *Nicotiana*. Based on both floral morphology and color, four convergent regimes were identified (Fig. 7). The floral characters of the ‘green’ regime and the ‘white, stellate’ regime largely correspond to traditionally recognized pollination syndromes [56, 57]. The ‘white, stellate’ regime includes diploid *N. alata*, *N. plumbaginifolia*, and *N. longiflora*, which are characterized by white flowers with long, narrow corolla tubes and stellate floral limb outlines (Fig. 7) that open at night [41]). This combination of traits is associated with hawkmoth pollination [45, 58, 59], and both *N. alata* and *N. longiflora* are primarily pollinated by hawkmoths [50]. *Nicotiana plumbaginifolia* is largely selfing [50]. The ‘green’ regime includes *N. rustica* (allotetraploid), *N. benavidesii*, *N. raimondii*, *N. solanifolia*, *N. paniculata*, *N. knightiana*, and *N. langsdorffii* (all diploids)*.* These species are characterized by flowers with medium corolla width, a round floral limb outline, a reduced floral limb compared to the floral tube opening, which is often reflexed at anthesis (Fig. 7), and often copious nectar (personal observation), which is consistent with a hummingbird pollination syndrome, although these flowers are green instead of the traditional red. In fact, hummingbirds are the primary pollinator for *N. langsdorffii* [50] and *N. paniculata* [60] and have been observed visiting *N. raimondii* (S. Knapp, personal observation); however, information on the pollinators of the other species is unavailable. Similarly, little is known about the pollinators of the other two convergent regimes, the ‘high UV’ regime and the ‘pink’ regime. However, one species of the ‘pink’ regime, *N. otophora*, is pollinated by bats [61]. The wide tubes and copious nectar found in the flowers of this regime are consistent with a bat pollination syndrome, but pink, magenta, and red flower colors are not usually associated with bat pollination. The ‘high UV’ regime has flowers with a round floral outline and a high ratio of UV reflectance compared to the human visible range. This high incidence of UV reflectance suggests that this regime may be associated with pollinators that have UV receptors, but the pollinators of these species are unknown. However, most of these species, except *N. cordifolia*, are UV-white; therefore, they may not be attractive to bees, which find it difficult to distinguish UV-white objects from the background material [62]. In addition, bees prefer highly dissected floral outlines to round floral outlines [63], suggesting that these flowers may perhaps be unlikely to be pollinated by bees.

## Conclusions

Our results show that using reconstructed progenitor phenotypes from the point of allopolyploid origin can potentially alter the interpretation of the progression of allopolyploid evolution, especially in older allopolyploids. We also show that young allopolyploids display different trends in floral evolution than older allopolyploids or diploids, suggesting that the early consequences of allopolyploidy can alter floral evolution, at least in *Nicotiana* allopolyploids. These young allopolyploids have shorter, wider corolla tubes, which suggests that a morphological change toward more generalist pollination may accompany allopolyploidy. Evolutionary lability of the specific floral traits observed here also differs among young allopolyploids, older allopolyploids, and diploids. Corolla tube length evolution is more labile in diploids and older allopolyploids than in young allopolyploids and seems to be associated with species divergence and sometimes differences in primary pollinator type. The presence of convergent regimes, some of which resemble traditional pollination syndromes, suggests that selection, perhaps pollinator-mediated, may be involved in shaping floral evolution in *Nicotiana*. Together, these results suggest that pollination may have influenced both allopolyploid and diploid evolution in *Nicotiana*, but further studies are needed to determine whether the divergence in floral phenotypes observed affects pollinator preference and efficiency in nature.

## Methods

### Plant material and growth conditions

For these analyses, we combined the datasets included in McCarthy et al. [20] with additional floral measurements that we obtained at the University of California, Riverside (UCR), USA to generate an expanded morphological dataset that increases sampling across the genus, including diploid and allotetraploid species as well as synthetic allotetraploids created from crosses between the same progenitor species as natural allotetraploids. Although these synthetic allopolyploids provide an excellent opportunity to compare the immediate consequences of allopolyploidy to the effects of subsequent allopolyploid evolution, it is important to note that the diploid individuals used to create these synthetic allopolyploids may differ genetically, and hence potentially phenotypically, from the actual progenitors of the natural allopolyploids, due both to intraspecific genetic variation and to continuing evolution of progenitor species since the time of natural allopolyploid origin. Plants were grown at 20 °C in a greenhouse exposed to natural light and watered with liquid fertilizer via a drip system. For several species, we used plant material from multiple origins; a list of the species and accessions included in our analyses can be found in Additional file 7: Table S2. TW and TH accessions come from the United States *Nicotiana* Germplasm Collection. 9-digit **4750*** accessions come from Radboud University, Nijmegen, The Netherlands. ‘Baldwin’ accessions come from the Baldwin lab (Max Planck Institute, Jena, Germany). CAM accessions come from the Cambridge University Botanic Garden, UK. CPG accessions come from the Chelsea Physic Garden, UK. The 095–55 accession comes from IPK Gatersleben, Germany. *Nicotiana ‘rastroensis’* material came from the Smith Lab (University of Colorado, Boulder, Boulder, CO, USA), voucher: Holtsford, *s.n.* and represents an undescribed species. 517** accessions were collected in Bolivia with a permit issued to Dr. Michael Nee of the New York Botanical Garden by the government of Bolivia for the year 2000. Specimens were identified by Dr. Michael Nee and Dr. Sandra Knapp, and voucher specimens were deposited in the Herbarium at the Natural History Museum, London, catalog numbers BM000940731, BM000940688, and BM000940685. *Nicotiana tabacum* ‘Chulumani’ was collected in 1950 by Winifred Mary Adelaide Brooke; the voucher specimen was deposited in the Natural History Museum, London, catalog number BM001070053, and identified by Dr. Sandra Knapp. The first and second generation synthetic *N. rustica* (S_0_ and S_1_, respectively) were from the same synthetic line; therefore, for analyses, we averaged their morphological data. We likewise averaged data for *N. stocktonii* TW126 and 974,750,101 because these accessions are from the same field collection; USDA (TW126) sent seed material from this collection to Nijmegen (974750101).

### DNA extraction, PCR, and sequencing

A well-supported phylogenetic tree provides the most accurate results for ancestral character state reconstruction; therefore, we performed a phylogenetic analysis of *Nicotiana* using a concatenated dataset from published sequence data to increase resolution and support for deep ancestral nodes (details in the next section), as well as increasing taxonomic sampling. Based on available GenBank sequences from previous analyses [36–39, 64], two nodes of allopolyploid origin for our study species were not well resolved with the allopolyploid sequence data available. To increase resolution and support for these nodes, we generated additional sequence data for two loci from three allotetraploid species, *N. quadrivalvis* 904750042, *N. nudicaulis* 964750114, and *N. repanda* TW110, for which only sequences from diploid species were available. We extracted genomic DNA using the Qiagen DNeasy Plant Mini Kit (Qiagen, Hilden, Germany) and amplified *GLOBOSA* (*GLO*; 3rd-6th exon) and *WAXY* (5th–9th exon) using 10 pmol primer (primer sequences from Kelly et al. ([39]; Table 4)) and EconoTaq (Lucigen) under the following conditions: initial denaturation at 94 °C for 3 min, followed by 35 cycles of 94 °C for 1 min, 52 °C (*GLO*) or 48 °C (*WAXY*) annealing temperature for 1 min, 72 °C extension for 1 (*GLO*) or 2 (*WAXY*) minutes, followed by a final 7 min extension at 72 °C. We cleaned PCR products using the Qiaquick PCR Purification Kit (Qiagen, Hilden, Germany) following the manufacturer’s protocols. In order to obtain sequences from both progenitor copies from these allopolyploid species, we cloned PCR products using the TOPO-TA Cloning Kit (Invitrogen, Carlsbad, CA, USA), selected and PCR screened white colonies with M13 primers, and performed plasmid preps using the Qiaprep Miniprep Spin Kit (Qiagen, Hilden, Germany). We sequenced the clones and added the resulting sequences to existing alignments for phylogenetic reconstruction.

**Table 4:** *GLOBOSA* and *WAXY* primer sequences

Primer	Sequence
GLO 358F	5′ - ATG ATG TTG GAA GAT GCC CTT G - 3′
GLO 600R	5′ - TAG GCT GCA TTG GCT GAA CTC - 3′
WAXY 181F	5′ - CGG GTA ATG ACA ATA TST CC - 3′
WAXY Nico F	5′ - GCT ACC TAA AGT CGA TGT ACC - 3′
WAXY Nico R	5′ - TGT TCC ATA GCG CAT AGC ATG - 3′

### Phylogenetic reconstruction from a concatenated dataset

For phylogenetic reconstruction, we used both the newly generated sequences described above and downloaded all available *Nicotiana* sequences for the following loci from GenBank: nuclear genes *ALCOHOL DEHYDROGENASE* (*ADH*)*, GLO, GLUTAMINE SYNTHETASE* (*GS*)*,* ITS*, LEAFY/FLORICAULA* (*LFY/FLO*)*, MADS1/FRUITFULL* (*MADS1/FUL*)*,* and *WAXY*, and the plastid genes and introns/spacers *trnL-F*, *trnS-G*, *matK*, *ndhF*, *trnH-R*, *rpoC1*, *trnK*, *accD*, and *rbcL*. GenBank accession numbers of all sequences are found in Additional file 1: Table S1.

Starting with alignments from previous studies, we independently aligned all loci manually as needed using Geneious (Biomatters) and concatenated these alignments using SequenceMatrix [65]. Total taxon sampling consisted of 41 of the 76 species currently recognized in *Nicotiana* [66, 67]: 32 diploid and nine allopolyploid species. The oldest allopolyploid section, section *Suaveolentes*, was not included in our analysis because its origin is less straightforward than those of the younger allopolyploid sections [40]. In addition, we did not include homoploid (diploid) hybrid species. Unlike allopolyploids, homoploid hybrid species do not maintain fixed heterozygosity; instead, they tend to retain only one progenitor copy per gene. The progenitor sequence that is retained, however, will vary from gene to gene, leading to incongruence in placement of homoploid hybrid species in single locus trees. This conflict among gene trees can result in poor support and spurious taxon placement in trees resulting from concatenated matrices. Therefore, we excluded documented homoploid hybrid species, *N. glauca, N. glutinosa, N. linearis,* and *N. spegazzinii* [38, 39], from our analyses, as well as *N. acaulis* due to previously observed inconsistencies in its placement among single locus trees [36–39]. For allopolyploid taxa, we included both maternal and paternal copies of each gene in the data matrix and distinguished between them with the addition of numbers to the species name (1 for maternal and 2 for paternal). Average gene coverage (the percentage of taxa included for a given locus) was 73.7%, with *GS* having the highest coverage (89.6%) and *MADS1/FUL* having the lowest coverage (60.3%; Table 5). The concatenated data matrix consisted of 919,706 cells, with 490,886 of these having nucleotide data (53.4% nucleotide coverage). Although we do not have complete data for all species, the supermatrix method we have used has been shown to yield accurate topologies even with large amounts of missing data [68–71]. We have deposited the alignment files of each locus and the concatenated dataset in Dryad (10.5061/dryad.f374gf0).

**Table 5:** Phylogenetic statistics

	*ADH*	*GLO*	*GS*	ITS	*LFY*	*MADS1/FUL*	*WAXY*	plastid	All
Number of taxa	44	36	53	46	44	35	39	45	62
Length (bp)	1057	1499	1549	794	1567	1566	1154	6662	15,857
Variable characters	323	418	556	209	555	370	293	461	3182
Parsimony informative characters	195	184	349	130	360	191	147	256	1812
Model of Evolution (AIC criterion)	TrN + G	TVM + G	GTR + G	GTR + I + G	TrN + G	TVM + G	HKY + G	TVM + G	NA
Likelihood score	− 4362. 376	− 5508. 128	− 7308. 117	− 3672. 139	− 7708. 691	− 5454. 690	− 4156. 594	−12,995. 787	−53,019. 488

We conducted phylogenetic analyses on the concatenated dataset using maximum likelihood (ML; Garli version 2.01; [72] and Bayesian inference (MrBayes, version 3.2.5; [73], whereas we analyzed individual loci using only ML. For each analysis, we chose taxa in *Anthocercis, Cyphanathera,* and *Symonanthus* as outgroups based on previous analyses of Solanaceae [36, 37, 74, 75]. Due to missing outgroup data at the species level, we created generic level outgroups by concatenating sequences from multiple species within *Anthocercis, Cyphanathera,* and *Symonanthus*.

We chose to use Garli for ML analysis because it allows for greater choice in models of molecular evolution compared to RAxML [76]. We analyzed each locus in jModelTest, version 2.1.2, [77]; Table 5) and selected appropriate models using Akaike information criterion (AIC) estimates [78]. For the ML analyses, we completed 20 independent runs for each locus (Additional file 8: Fig. S6) and the concatenated full dataset, using the single-threaded instance of Garli on the University of Florida High Performance Computing Cluster with appropriate models of evolution for each partition in the analysis. We set each ML search to terminate if there were no improvements to the likelihood value of the tree after 10,000 generations. In addition, we conducted ML bootstrap analysis with 1000 replicates on each individual gene, the plastid-only dataset, and the full dataset. We conducted MrBayes runs on the concatenated dataset with appropriate models of evolution for 5,000,000 generations with two independent runs with four chains sampled every 1000 generations until the split deviations were less than 0.004. Removal of the post-burn in of 25% was conducted before a consensus tree with posterior probabilities was generated.

### Floral character measurements

For all species included in this study, we photographed flowers and analyzed the resulting images as described in McCarthy et al. [20]. Briefly, we took five front (depicting the floral limb—the lobed and spreading portion of the corolla) and dissected (cut along the floral tube at a midrib and pinned open) view photographs per flower, five flowers per plant, and five plants per accession (where available). For geometric morphometric analysis of floral limb shape, we assigned 15 landmarks to photographs [20] and used TPS software [79–81] to analyze floral shape independent of floral size. We measured corolla tube length and width from dissected and front view photographs using ImageJ, version 1.51 k, [82]. We used flower and species averages of tube length and width and PC1 (principal component 1) and PC2 produced by the geometric morphometric analysis in subsequent analyses. We analyzed PC1/PC2 as a two-dimensional trait but tube length and width separately because tube length and width are not genetically linked [83, 84].

### Continuous character ancestral state reconstructions

We performed ancestral state reconstructions of continuous characters for PC1, PC2, tube length, and tube width using anc.ML in the phytools package, version 0.5–38, [85] in R, version 3.4.2, [86]. R scripts are uploaded to GitHub (https://github.com/elizabethwmccarthy/Ancestral-reconstruction). For analysis of trait evolution across diploids, we used a tree with only diploid species. We used cont.ML in the phytools package [85] to produce heat maps of character evolution for tube length and width. Because we analyzed floral shape as a single, two-dimensional trait, PC1 and PC2 coordinates from ancestral nodes were used to extract splines from the morphospace to represent reconstructed phenotypes. We identified shifts in morphology for each trait (shape, tube length, and tube width) by calculating the difference between the values for each reconstructed trait for successive nodes (e.g. between internal nodes connected by a branch or between an extant taxon and the node of its most recent common ancestor). We considered a shift in morphology to have occurred only if the difference between the reconstructed values for these successive nodes was greater than 10% of the range observed in extant species for that trait *(*e.g. if the range of tube length observed in extant species was 10 cm, then shifts in morphology would occur when the difference between successive nodes was greater than 1 cm). We chose this threshold because the median intraspecific variation across all traits was 10% of the total range.

For estimating ancestral characters at nodes arising from polyploid formation (circled in red in Fig. 1), we used the ML tree that includes both diploid and allopolyploid species. We used only diploid morphology, however, to estimate ancestral character states because we want to reconstruct ancestral phenotypes based on the bifurcating evolution of diploid species. We plotted the reconstructed progenitor nodes for each dataset (PC1, PC2, tube length, and tube width) along with the data for extant progenitor morphology to visualize the difference between reconstructed values and observed extant morphology.

### Analysis of diploid divergence

In order to compare trends in morphological evolution between diploids and allopolyploids, we used the values from the ancestral character state reconstructions on the diploid-only tree to examine trends in diploid divergence. Diploid divergence can be measured across the entire tree based on the difference between reconstructed values at successive nodes of the tree. We visualized the direction of these differences by plotting the values for the reconstructed nodes in the floral trait morphospaces along with the data for extant species. We connected successive nodes (from older to more recent) with arrows (vectors) in order to recapitulate the progression of evolution through the morphospace based on phylogenetic relationships.

We examined trends in the morphological evolution of diploids by mathematically translating these vectors to the origin of a graph, with the older node at the origin. We used a modification of the Rayleigh test [87], introduced by Moore [88], which takes into account the magnitude as well as the direction of the change, to determine whether these vectors were uniformly distributed around the origin. We designate this test as ‘Moore-Rayleigh’ in subsequent occurrence in the text and determined significance based on the probability distribution in Moore [88] after Bonferroni correction. R scripts are uploaded to GitHub (https://github.com/elizabethwmccarthy/Ancestral-reconstruction).

In addition, we tested whether individual traits showed phylogenetic signal using Blomberg’s K and Pagel’s λ in order to determine whether the evolution of floral traits followed the trajectory of phylogenetic divergence, or whether more complex patterns were apparent. We implemented these analyses in R using the phytools package [85]. R scripts are uploaded to GitHub (https://github.com/elizabethwmccarthy/Ancestral-reconstruction).

### Analyses of allopolyploid divergence using extant versus reconstructed progenitor phenotypes

For analysis of morphological evolution in allopolyploids, we followed the methods described in McCarthy et al. [20]. Briefly, the simplest null hypothesis is that allopolyploids will display a phenotype intermediate between those of their progenitors. Therefore, for each allopolyploid accession and floral trait, we found the mean for each progenitor species and then took the average of the two to estimate the expected allopolyploid morphology. We designated this value the progenitor midpoint. Although allopolyploids may display phenotypes that are intermediate, like one or the other parent, or transgressive, we chose to use intermediacy because the average of the progenitors is the simplest null hypothesis for the expected allopolyploid phenotype. This allows us to consistently and unbiasedly calculate an estimation of the direction and magnitude of evolution in allopolyploids. In contrast, it would be impossible to decide on one of the infinite number of possible phenotypes if we used a transgressive phenotype as our expectation following allopolyploid origin, and therefore, we would not be able to perform the analyses we present here.

We determined whether there were trends in the morphological evolution of allopolyploids by examining the direction in which they differed from their progenitor midpoints. We mathematically translated all midpoints to the origin of a graph and plotted the direction and magnitude of the distance between each allopolyploid accession and its progenitor midpoint accordingly. This allowed us to examine whether trends in the morphological evolution of allopolyploids were uniformly distributed in this space using the Moore-Rayleigh test [87, 88] as described above.

We performed the above analyses using both extant and reconstructed progenitor phenotypes and compared the results from each to determine whether our interpretations of the morphological evolution of allopolyploids differed based on using extant versus reconstructed progenitor phenotypes. We tested the entire polyploid dataset, as well as young (synthetic to 0.7 million year old (myo) that have not speciated subsequent to allopolyploid origin) and older (1.4–4.3 myo, in which subsequent speciation has occurred) allopolyploids separately for each morphological dataset (floral limb shape and corolla tube length and width). The presence or absence of speciation subsequent to allopolyploid origin was based on the current taxonomy of *Nicotiana* species [66]. We compared the Moore-Rayleigh test results for diploids and allopolyploids to determine whether trends in the morphological evolution of allopolyploids differed from that of diploids, applying Bonferroni correction to determine significance. We also calculated the difference between extant and reconstructed progenitor phenotypes in all traits and plotted these values against allopolyploid age to determine whether this difference increases over time. Parentage and age of the allopolyploid species examined here can be found in Table 1.

### Spectral reflectance measurements, normalization, and principal components analysis (PCA)

We quantified floral color using spectral reflectance measurements. Most spectra used here have been previously published [31, 32, 89], but we obtained new spectra for *N. alata*, *N. bonariensis*, *N. cordifolia*, *N. corymbosa*, *N. forgetiana*, *N. kawakamii*, *N. longiflora*, and *N. solanifolia* using a JAZ spectrophotometer with a pulsed xenon light source (Ocean Optics, Dunedin, Florida, USA) and standardized using a Spectralon white standard (Labsphere, North Sutton, New Hampshire, USA). We smoothed spectra three times with a rolling average over 9 nm [31]. In order to compare spectra based on their shape instead of their overall intensity, we normalized spectra to the same area under the curve from 300 to 700 nm. We then performed PCA on the normalized spectral values in 25 nm increments (300 nm, 325 nm, 350 nm, etc), using the ade4 package, version 1.7.11, in R [90] and used the resulting first three principal components (the only three with > 5% of the variation), which accounted for 91.73% of the variation present in the dataset, in further analyses (Additional file 9: Figure S7). R scripts are uploaded to GitHub (https://github.com/elizabethwmccarthy/Ancestral-reconstruction).

### Convergent evolution analyses

To determine whether polyploids display novel suites of floral characters or share the same suites of characters as diploid species, we ran analyses to test for convergent evolution using the surface package, version 0.4.1, [91] in R. R scripts are uploaded to GitHub (https://github.com/elizabethwmccarthy/Ancestral-reconstruction). Briefly, this package uses data from multiple continuous traits and a phylogenetic tree and applies a Hansen model of stabilizing selection around multiple adaptive peaks in order to identify evolutionary shifts in a phylogenetic context. In addition, the analysis then determines whether any of these shifts represent convergent regimes using maximum likelihood and AIC to assess whether convergent regimes improve the fit of the model. If the collapse of two independent shifts into a single regime improves the fit, these shifts are deemed convergent. We ran three sets of analyses: only morphological data (the first two principal components of the geometric morphometric analysis of floral shape, tube length, and tube width), only floral color data (the first three principal components of the spectral reflectance PCA), and both morphological and color data (all seven of these traits). We used the diploid + allopolyploid tree with both allopolyploid homeologs in order to evaluate suites of floral characters in the context of both maternal and paternal phenotypes. We also added polytomies to the tree if we had multiple accessions of allopolyploid species for our floral traits datasets, but only one accession included in the tree to incorporate variation among allopolyploid accession of the same species in our analyses.

## Additional files

Additional file 1: **Table S1.** GenBank accession numbers for all sequences used. (XLSX 15 kb)

Additional file 2: **Figure S1.** Morphospace for floral limb shape and corolla tube length and width datasets. (PPTX 327 kb)

Additional file 3: **Figure S2.** Extant and reconstructed progenitor midpoints for floral limb shape. (PPTX 198 kb)

Additional file 4: **Figure S3.** Extant and reconstructed progenitor midpoints for corolla tube length and width. (PPTX 171 kb)

Additional file 5: **Figure S4.** Convergent regimes based on only morphological characters. (PPTX 607 kb)

Additional file 6: **Figure S5.** Convergent regimes based on only color characters. (PPTX 488 kb)

Additional file 7: **Table S2.** *Nicotiana* species and accessions analyzed for floral morphology. (DOCX 19 kb)

Additional file 8: **Figure S6.** Maximum likelihood trees for individual loci. (PPTX 1505 kb)

Additional file 9: **Figure S7.** Spectra principal components analysis. (PPTX 45 kb)

## Abbreviations

*ADH*: *ALCOHOL DEHYDROGENASE*
*GLO*: *GLOBOSA*
*GS*: *GLUTAMINE SYNTHETASE*
*ITS*: *INTERNAL TRANSCRIBED SPACER*
*LFY/FLO*: *LEAFY/FLORICAULA*
*MADS1/FUL*: *MADS1/FRUITFULL*
ML: maximum likelihood
myo: million years old
PC: principal component
PCA: principal components analysis
PCR: polymerase chain reaction
UCR: University of California, Riverside
