# Supplementary material for: Early consequences of allopolyploidy alter floral evolution in Nicotiana (Solanaceae)
Source: BMC Plant Biol. 2019 Apr 27;19:162. doi: 10.1186/s12870-019-1771-5 (PMC6486959; doi:10.1186/s12870-019-1771-5)
Supplement: Supplementary file 2 — Figure S1. Morphospace for floral limb shape and corolla tube length and width datasets. (PPTX 327 kb) [file 12870_2019_1771_MOESM2_ESM.pptx]

## Slide 1
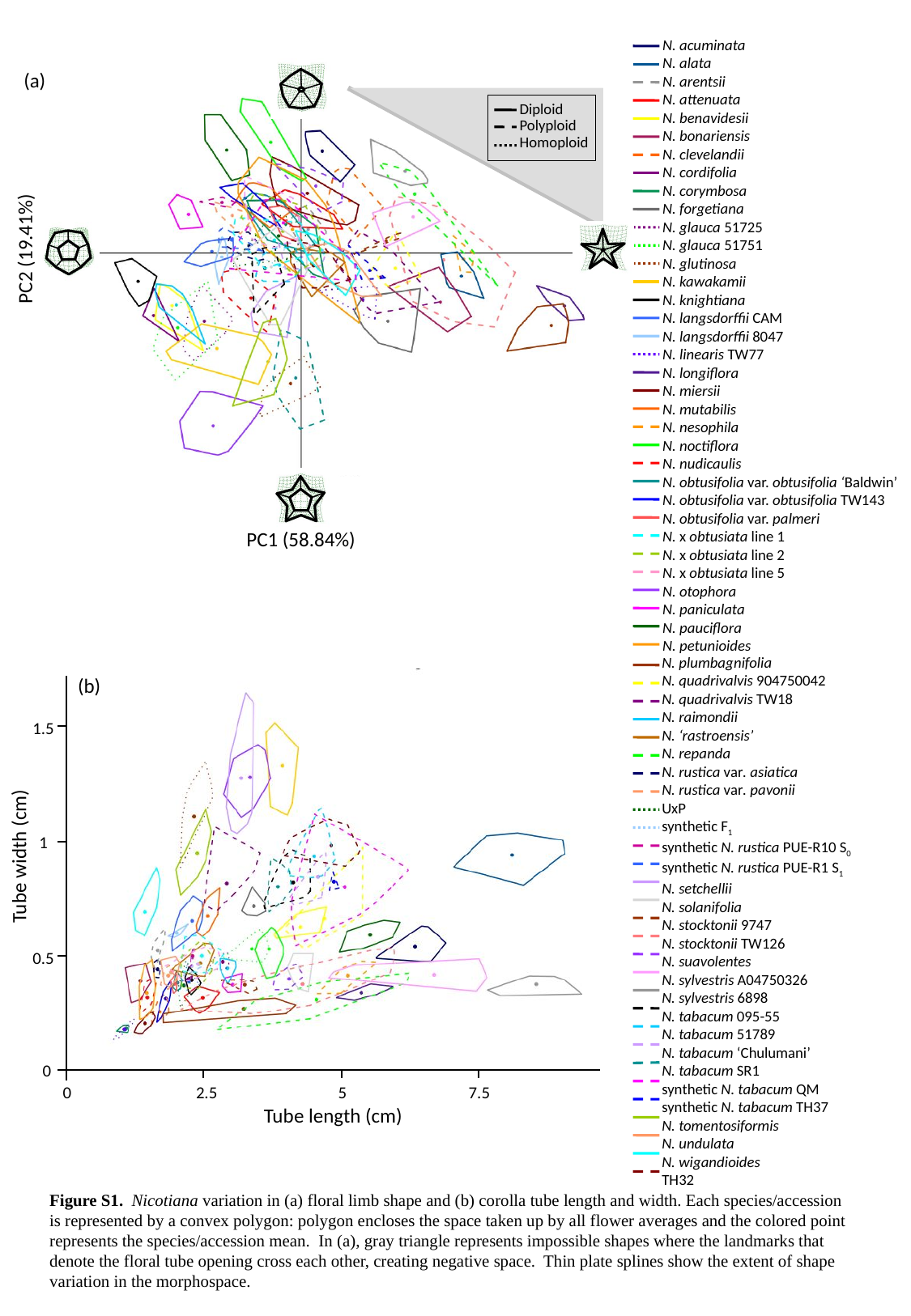

N. acuminata
N. alata
N. arentsii
N. attenuata
N. benavidesii
N. bonariensis
N. clevelandii
N. cordifolia
N. corymbosa
N. forgetiana
N. glauca 51725
N. glauca 51751
N. glutinosa
N. kawakamii
N. knightiana
N. langsdorffii CAM
N. langsdorffii 8047
N. linearis TW77
N. longiflora
N. miersii
N. mutabilis
N. nesophila
N. noctiflora
N. nudicaulis
N. obtusifolia var. obtusifolia ‘Baldwin’
N. obtusifolia var. obtusifolia TW143
N. obtusifolia var. palmeri
N. x obtusiata line 1
N. x obtusiata line 2
N. x obtusiata line 5
N. otophora
N. paniculata
N. pauciflora
N. petunioides
(a)
Diploid
Polyploid
Homoploid
PC2 (19.41%)
PC1 (58.84%)
N. plumbagnifolia
N. quadrivalvis 904750042
N. quadrivalvis TW18
N. raimondii
N. ‘rastroensis’
N. repanda
N. rustica var. asiatica
N. rustica var. pavonii
UxP
synthetic F1
synthetic N. rustica PUE-R10 S0
synthetic N. rustica PUE-R1 S1
N. setchellii
N. solanifolia
N. stocktonii 9747
N. stocktonii TW126
N. suavolentes
N. sylvestris A04750326
N. sylvestris 6898
N. tabacum 095-55
N. tabacum 51789
N. tabacum ‘Chulumani’
N. tabacum SR1
synthetic N. tabacum QM
synthetic N. tabacum TH37
N. tomentosiformis
N. undulata
N. wigandioides
TH32
(b)
1.5
1
Tube width (cm)
0.5
0
0
2.5
5
7.5
Tube length (cm)
Figure S1. Nicotiana variation in (a) floral limb shape and (b) corolla tube length and width. Each species/accession is represented by a convex polygon: polygon encloses the space taken up by all flower averages and the colored point represents the species/accession mean. In (a), gray triangle represents impossible shapes where the landmarks that denote the floral tube opening cross each other, creating negative space. Thin plate splines show the extent of shape variation in the morphospace.
