# Supplementary material for: Early consequences of allopolyploidy alter floral evolution in Nicotiana (Solanaceae)
Source: BMC Plant Biol. 2019 Apr 27;19:162. doi: 10.1186/s12870-019-1771-5 (PMC6486959; doi:10.1186/s12870-019-1771-5)
Supplement: Supplementary file 3 — Figure S2. Extant and reconstructed progenitor midpoints for floral limb shape. (PPTX 198 kb) [file 12870_2019_1771_MOESM3_ESM.pptx]

## Slide 1
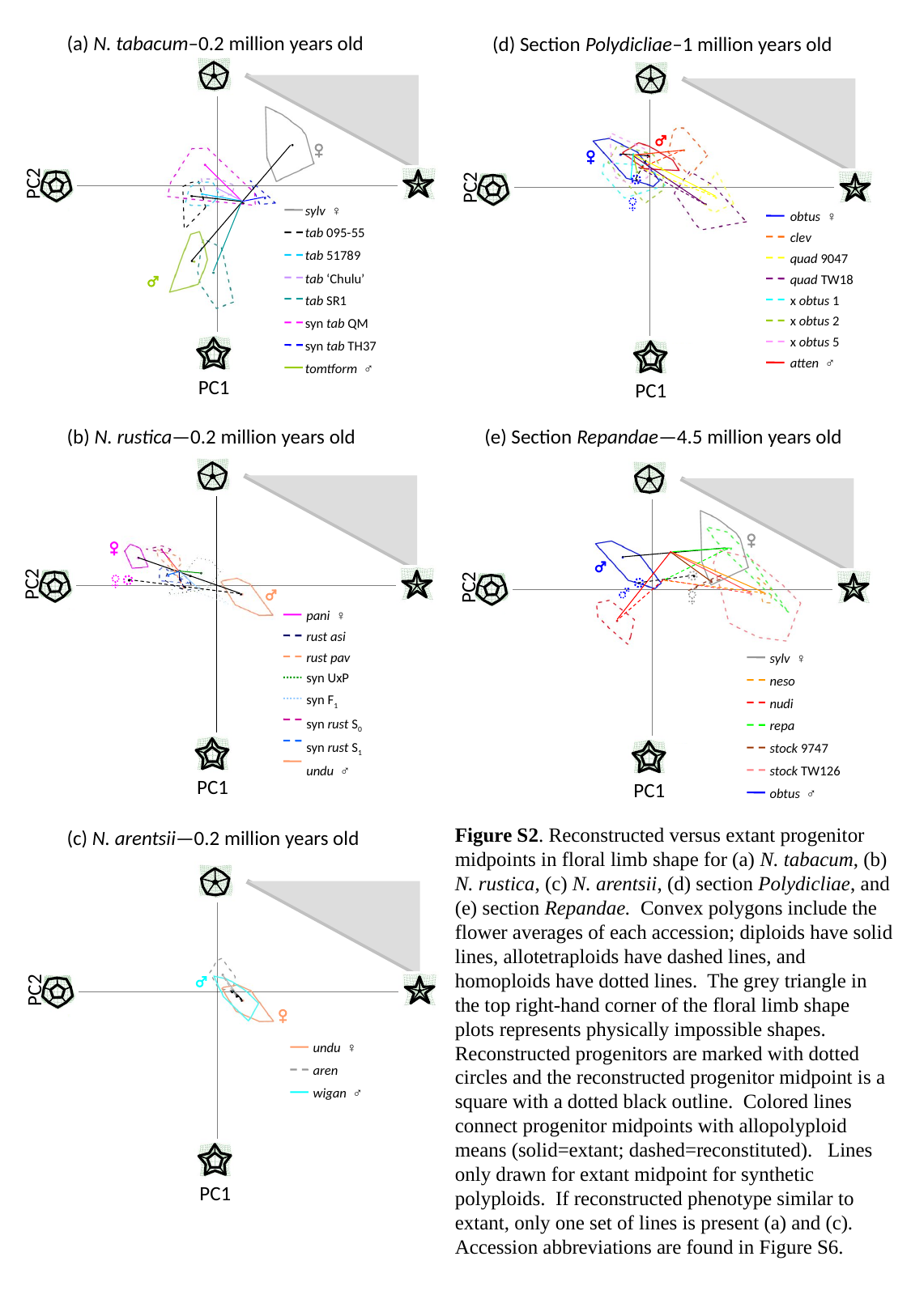

(a) N. tabacum–0.2 million years old
(d) Section Polydicliae–1 million years old
PC2
PC1
PC2
PC1
sylv ♀
tab 095-55
tab 51789
tab ‘Chulu’
tab SR1
syn tab QM
syn tab TH37
tomtform ♂
obtus ♀
clev
quad 9047
quad TW18
x obtus 1
x obtus 2
x obtus 5
atten ♂
(b) N. rustica—0.2 million years old
(e) Section Repandae—4.5 million years old
PC2
PC1
PC2
PC1
pani ♀
rust asi
rust pav
syn UxP
syn F1
syn rust S0
syn rust S1
undu ♂
sylv ♀
neso
nudi
repa
stock 9747
stock TW126
obtus ♂
Figure S2. Reconstructed versus extant progenitor midpoints in floral limb shape for (a) N. tabacum, (b) N. rustica, (c) N. arentsii, (d) section Polydicliae, and (e) section Repandae. Convex polygons include the flower averages of each accession; diploids have solid lines, allotetraploids have dashed lines, and homoploids have dotted lines. The grey triangle in the top right-hand corner of the floral limb shape plots represents physically impossible shapes. Reconstructed progenitors are marked with dotted circles and the reconstructed progenitor midpoint is a square with a dotted black outline. Colored lines connect progenitor midpoints with allopolyploid means (solid=extant; dashed=reconstituted). Lines only drawn for extant midpoint for synthetic polyploids. If reconstructed phenotype similar to extant, only one set of lines is present (a) and (c). Accession abbreviations are found in Figure S6.
(c) N. arentsii—0.2 million years old
PC2
PC1
undu ♀
aren
wigan ♂
