# Supplementary material for: Early consequences of allopolyploidy alter floral evolution in Nicotiana (Solanaceae)
Source: BMC Plant Biol. 2019 Apr 27;19:162. doi: 10.1186/s12870-019-1771-5 (PMC6486959; doi:10.1186/s12870-019-1771-5)
Supplement: Supplementary file 4 — Figure S3. Extant and reconstructed progenitor midpoints for corolla tube length and width. (PPTX 171 kb) [file 12870_2019_1771_MOESM4_ESM.pptx]

## Slide 1
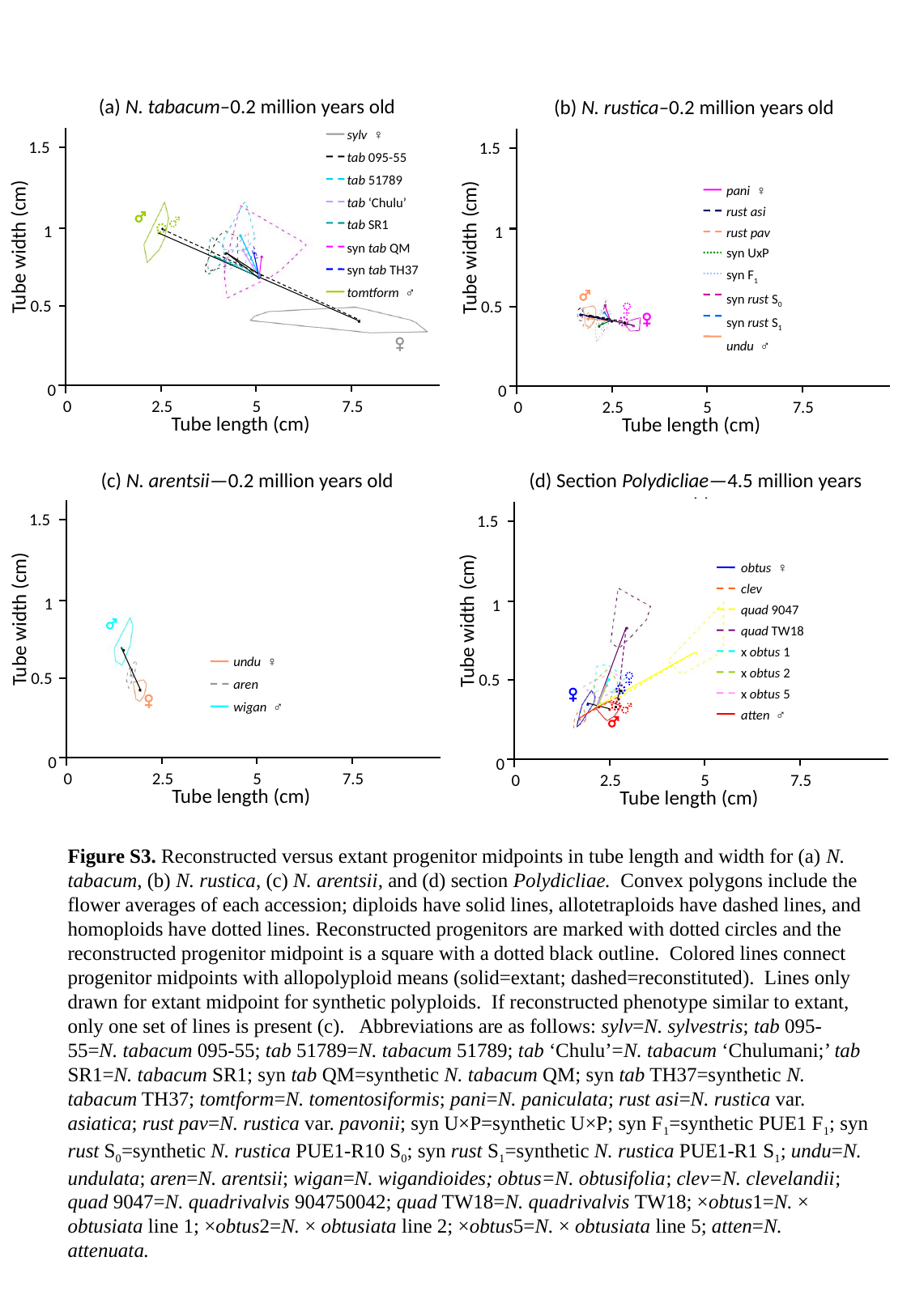

(a) N. tabacum–0.2 million years old
1.5
1
Tube width (cm)
0.5
0
0
2.5
5
7.5
Tube length (cm)
(b) N. rustica–0.2 million years old
1.5
1
Tube width (cm)
0.5
0
0
2.5
5
7.5
Tube length (cm)
sylv ♀
tab 095-55
tab 51789
tab ‘Chulu’
tab SR1
syn tab QM
syn tab TH37
tomtform ♂
pani ♀
rust asi
rust pav
syn UxP
syn F1
syn rust S0
syn rust S1
undu ♂
(c) N. arentsii—0.2 million years old
1.5
1
Tube width (cm)
0.5
0
0
2.5
5
7.5
Tube length (cm)
(d) Section Polydicliae—4.5 million years old
1.5
1
Tube width (cm)
0.5
0
0
2.5
5
7.5
Tube length (cm)
obtus ♀
clev
quad 9047
quad TW18
x obtus 1
x obtus 2
x obtus 5
atten ♂
undu ♀
aren
wigan ♂
Figure S3. Reconstructed versus extant progenitor midpoints in tube length and width for (a) N. tabacum, (b) N. rustica, (c) N. arentsii, and (d) section Polydicliae. Convex polygons include the flower averages of each accession; diploids have solid lines, allotetraploids have dashed lines, and homoploids have dotted lines. Reconstructed progenitors are marked with dotted circles and the reconstructed progenitor midpoint is a square with a dotted black outline. Colored lines connect progenitor midpoints with allopolyploid means (solid=extant; dashed=reconstituted). Lines only drawn for extant midpoint for synthetic polyploids. If reconstructed phenotype similar to extant, only one set of lines is present (c). Abbreviations are as follows: sylv=N. sylvestris; tab 095-55=N. tabacum 095-55; tab 51789=N. tabacum 51789; tab ‘Chulu’=N. tabacum ‘Chulumani;’ tab SR1=N. tabacum SR1; syn tab QM=synthetic N. tabacum QM; syn tab TH37=synthetic N. tabacum TH37; tomtform=N. tomentosiformis; pani=N. paniculata; rust asi=N. rustica var. asiatica; rust pav=N. rustica var. pavonii; syn U×P=synthetic U×P; syn F1=synthetic PUE1 F1; syn rust S0=synthetic N. rustica PUE1-R10 S0; syn rust S1=synthetic N. rustica PUE1-R1 S1; undu=N. undulata; aren=N. arentsii; wigan=N. wigandioides; obtus=N. obtusifolia; clev=N. clevelandii; quad 9047=N. quadrivalvis 904750042; quad TW18=N. quadrivalvis TW18; ×obtus1=N. × obtusiata line 1; ×obtus2=N. × obtusiata line 2; ×obtus5=N. × obtusiata line 5; atten=N. attenuata.
