# Supplementary material for: Early consequences of allopolyploidy alter floral evolution in Nicotiana (Solanaceae)
Source: BMC Plant Biol. 2019 Apr 27;19:162. doi: 10.1186/s12870-019-1771-5 (PMC6486959; doi:10.1186/s12870-019-1771-5)
Supplement: Supplementary file 5 — Figure S4. Convergent regimes based on only morphological characters. (PPTX 607 kb) [file 12870_2019_1771_MOESM5_ESM.pptx]

## Slide 1
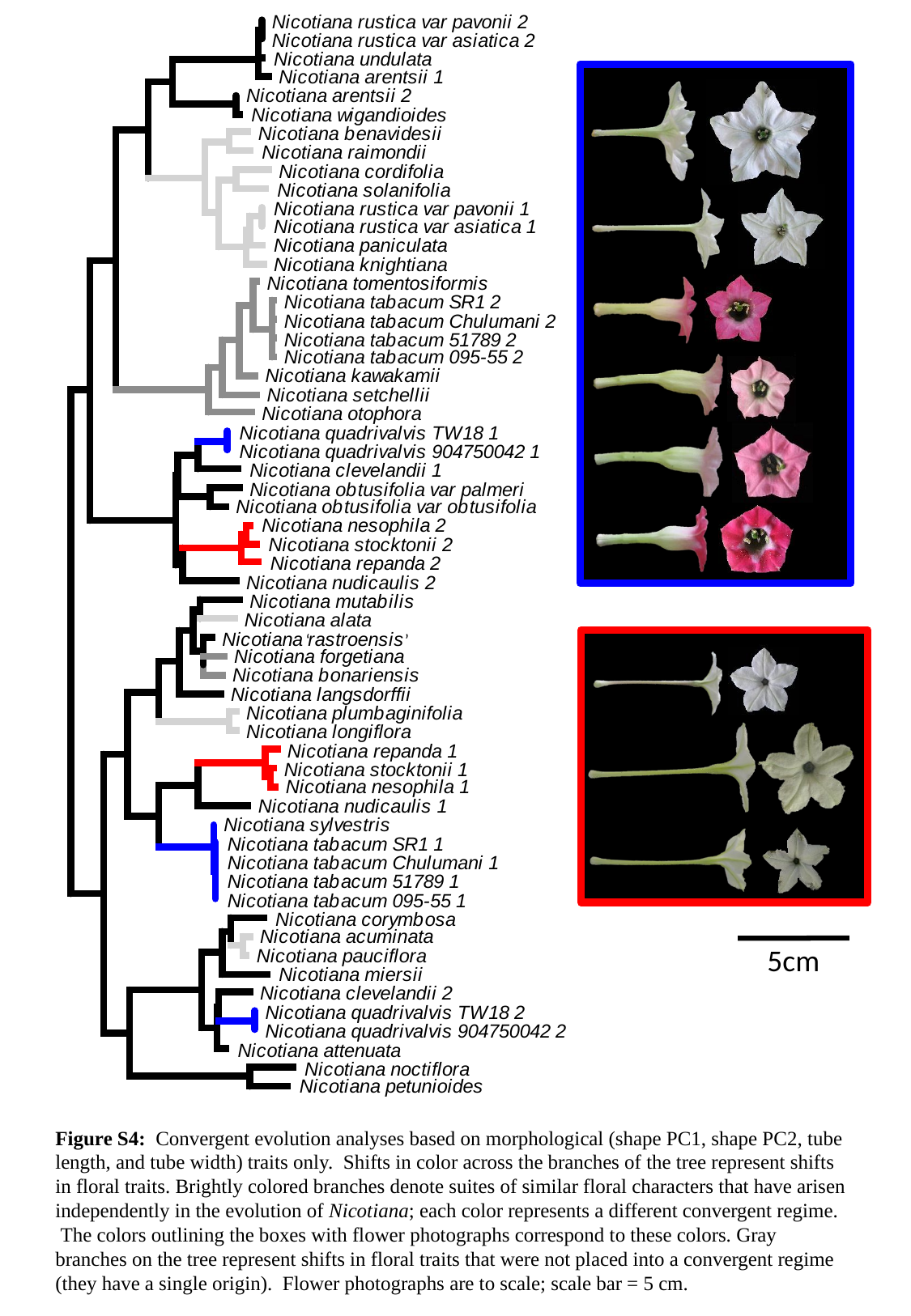

‘
’
5cm
Figure S4: Convergent evolution analyses based on morphological (shape PC1, shape PC2, tube length, and tube width) traits only.  Shifts in color across the branches of the tree represent shifts in floral traits. Brightly colored branches denote suites of similar floral characters that have arisen independently in the evolution of Nicotiana; each color represents a different convergent regime.  The colors outlining the boxes with flower photographs correspond to these colors. Gray branches on the tree represent shifts in floral traits that were not placed into a convergent regime (they have a single origin).  Flower photographs are to scale; scale bar = 5 cm.
