# Supplementary material for: Early consequences of allopolyploidy alter floral evolution in Nicotiana (Solanaceae)
Source: BMC Plant Biol. 2019 Apr 27;19:162. doi: 10.1186/s12870-019-1771-5 (PMC6486959; doi:10.1186/s12870-019-1771-5)
Supplement: Supplementary file 6 — Figure S5. Convergent regimes based on only color characters. (PPTX 488 kb) [file 12870_2019_1771_MOESM6_ESM.pptx]

## Slide 1
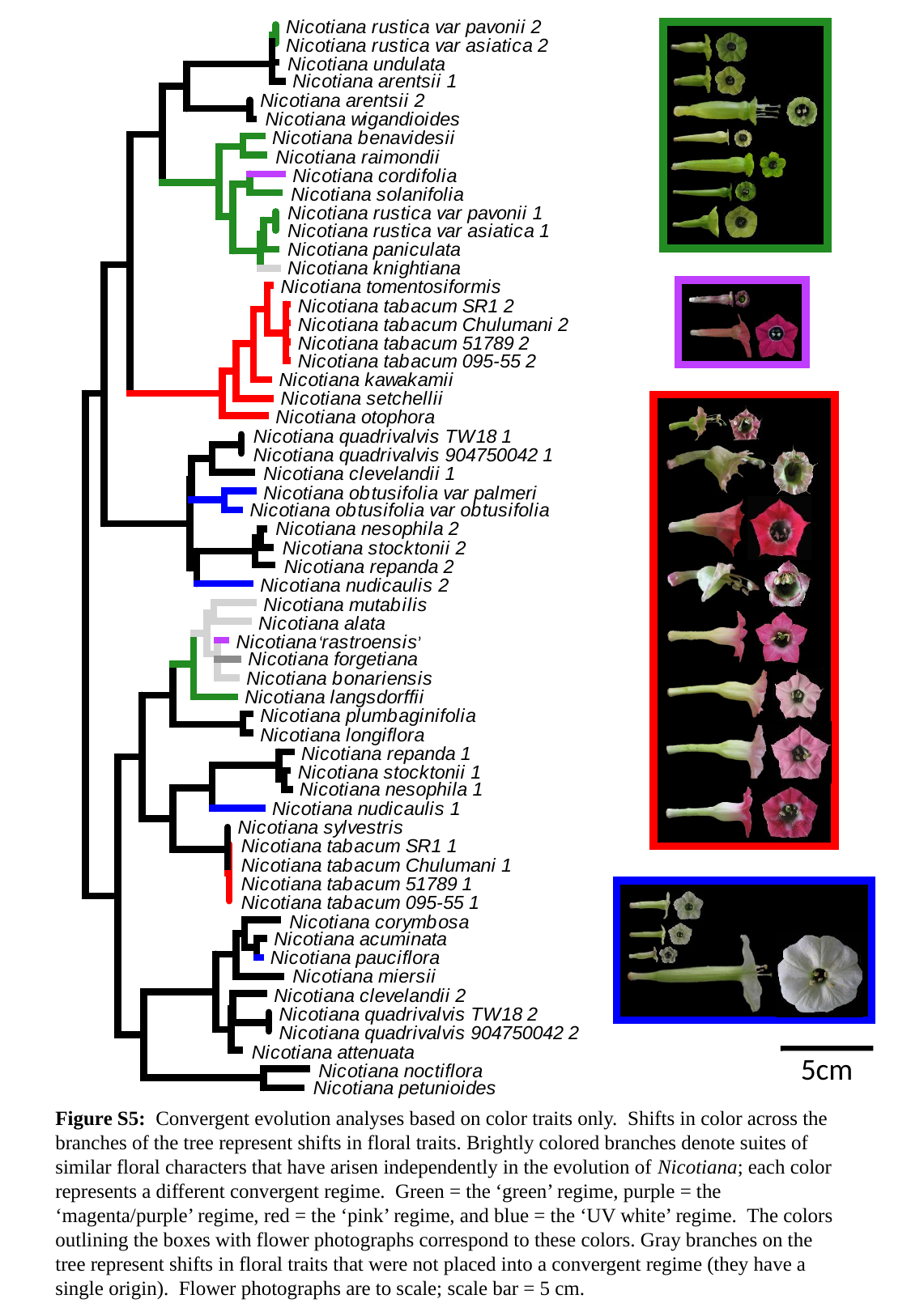

‘
’
5cm
Figure S5: Convergent evolution analyses based on color traits only.  Shifts in color across the branches of the tree represent shifts in floral traits. Brightly colored branches denote suites of similar floral characters that have arisen independently in the evolution of Nicotiana; each color represents a different convergent regime. Green = the ‘green’ regime, purple = the ‘magenta/purple’ regime, red = the ‘pink’ regime, and blue = the ‘UV white’ regime.  The colors outlining the boxes with flower photographs correspond to these colors. Gray branches on the tree represent shifts in floral traits that were not placed into a convergent regime (they have a single origin).  Flower photographs are to scale; scale bar = 5 cm.
