# Supplementary material for: Early consequences of allopolyploidy alter floral evolution in Nicotiana (Solanaceae)
Source: BMC Plant Biol. 2019 Apr 27;19:162. doi: 10.1186/s12870-019-1771-5 (PMC6486959; doi:10.1186/s12870-019-1771-5)
Supplement: Supplementary file 7 — Table S2. Nicotiana species and accessions analyzed for floral morphology. (DOCX 19 kb) [file 12870_2019_1771_MOESM7_ESM.docx]

**Table S2.** *Nicotiana* species and accessions analyzed for floral morphology

| **Species** | **Section** | **Ploidy** | **No. of plants** | **No. of flowers**^a^ | **QMUL or UCR?** |
| --- | --- | --- | --- | --- | --- |
| *N. acuminata* TW2 | *Petunioides* | diploid | 5 | 25 | QMUL |
| *N. alata* TW8 | *Alatae* | diploid | 5 | 25 | UCR |
| *N. arentsii* TW12 | *Undulatae* | tetraploid | 5 | 25 | QMUL |
| *N. attenuata ‘*Baldwin’ | *Petunioides* | diploid | 5 | 25 | both |
| *N. benavidesii* 894750181 | *Paniculatae* | diploid | 5 | 23 | QMUL |
| *N. bonariensis* TW28 | *Alatae* | diploid | 5 | 25 | both |
| *N. clevelandii* TW30 | *Polydicliae* | tetraploid | 5 | 25 | QMUL |
| *N. cordifolia* TW33 | *Paniculatae* | diploid | 1 | 5 | UCR |
| *N. corymbosa* TW35 | *Petunioides* | diploid | 5 | 5 | UCR |
| *N. forgetiana* TW50 | *Alatae* | diploid | 5 | 5 | UCR |
| *N. glauca* 51725 | *Noctiflorae-Petunioides* | homoploid | 4 | 20 | QMUL |
| *N. glauca* 51751 | *Noctiflorae-Petunioides* | homoploid | 5 | 25 | QMUL |
| *N. glutinosa* SCR1 1996 | *Tomentosae-Undulatae* | homoploid | 3 | 15 | QMUL |
| *N. kawakamii* TW72 | *Tomentosae* | diploid | 5 | 5 | UCR |
| *N. knightiana* CPG | *Paniculatae* | diploid | 5 | 25 | QMUL |
| *N. langsdorffii* 804750066 | *Alatae* | diploid | 1 | 5 | QMUL |
| *N. langsdorffii* CAM | *Alatae* | diploid | 5 | 25 | QMUL |
| *N. linearis* TW77 | *Noctiflorae-Petunioides* | homoploid | 2 | 10 | both |
| *N. longiflora* TW80 | *Alatae* | diploid | 5 | 25 | UCR |
| *N. miersii* TW85 | *Petunioides* | diploid | 10 | 50 | QMUL |
| *N. mutabilis* QMUL | *Alatae* | diploid | 1 | 5 | QMUL |
| *N. mutabilis* CPG12456 | *Alatae* | diploid | 5 | 25 | QMUL |
| *N. mutabilis* CPG3 | *Alatae* | diploid | 1 | 5 | QMUL |
| *N. nesophila* 974750097 | *Repandae* | tetraploid | 1 | 5 | QMUL |
| *N. noctiflora* TW88 | *Noctiflorae* | diploid | 3 | 15 | QMUL |
| *N. nudicaulis* 964750114 | *Repandae* | tetraploid | 5 | 25 | QMUL |
| *N.* × *obtusiata* line 1 ‘Baldwin’ | synthetic *Polydicliae* | tetraploid | 5 | 25 | QMUL |
| *N.* × *obtusiata* line 2 ‘Baldwin’ | synthetic *Polydicliae* | tetraploid | 5 | 25 | both |
| *N.* × *obtusiata* line 5 ‘Baldwin’ | synthetic *Polydicliae* | tetraploid | 5 | 25 | both |
| *N. obtusifolia* var. *obtusifolia* ‘Baldwin’ | *Trigonophyllae* | diploid | 5 | 25 | UCR |
| *N. obtusifolia* var. *obtusifolia* TW143 | *Trigonophyllae* | diploid | 5 | 25 | QMUL |
| *N. obtusifolia* var. *palmeri* TW98 | *Trigonophyllae* | diploid | 5 | 25 | QMUL |
| *N. otophora* TW95 | *Tomentosae* | diploid | 5 | 25 | both |
| *N. paniculata* | *Paniculatae* | diploid | 5 | 25 | QMUL |
| *N. pauciflora* TW104 | *Petunioides* | diploid | 5 | 25 | QMUL |
| *N. petunioides* TW105 | *Noctiflorae* | diploid | 5 | 29 | QMUL |
| *N. plumbaginifolia* TW106 | *Alatae* | diploid | 5 | 25 | QMUL |
| *N. quadrivalvis* 904750042 | *Polydicliae* | tetraploid | 5 | 25 | QMUL |
| *N. quadrivalvis* TW18 | *Polydicliae* | tetraploid | 5 | 25 | QMUL |
| *N. raimondii* TW109 | *Paniculatae* | diploid | 5 | 25 | QMUL |
| *N. ‘rastroensis’* | *Alatae* | diploid | 4 | 20 | UCR |
| *N. repanda* TW110 | *Repandae* | tetraploid | 5 | 25 | QMUL |
| *N. rustica* var. *asiatica* | *Rusticae* | tetraploid | 5 | 25 | QMUL |
| *N. rustica* var. *pavonii* | *Rusticae* | tetraploid | 5 | 25 | QMUL |
| Synthetic (U×P) | synthetic *Rusticae* | homoploid | 1 | 10 | QMUL |
| Synthetic PUE1 F_1_ | synthetic *Rusticae* | homoploid | 1 | 5 | QMUL |
| Synthetic *N. rustica* PUE1-R10 S_0_ | synthetic *Rusticae* | tetraploid | 1 | 5 | QMUL |
| Synthetic *N. rustica* PUE1-R1 S_1_ | synthetic *Rusticae* | tetraploid | 1 | 5 | QMUL |
| *N. setchellii* | *Tomentosae* | diploid | 5 | 25 | QMUL |
| *N. solanifolia* TW123 | *Paniculatae* | diploid | 5 | 25 | UCR |
| *N. stocktonii* 974750101 | *Repandae* | tetraploid | 1 | 5 | QMUL |
| *N. stocktonii* TW126 | *Repandae* | tetraploid | 5 | 25 | QMUL |
| *N. suaveolens* CAM | *Suaveolentes* | tetraploid | 5 | 25 | QMUL |
| *N. sylvestris* 6898 | *Sylvestres* | diploid | 5 | 25 | QMUL |
| *N. sylvestris* A04750326 | *Sylvestres* | diploid | 5 | 26 | both |
| *N. tabacum* 095-55 | *Nicotiana* | tetraploid | 5 | 25 | QMUL |
| *N. tabacum* 51789 | *Nicotiana* | tetraploid | 2 | 10 | QMUL |
| *N. tabacum* ‘Chulumani’ | *Nicotiana* | tetraploid | 4 | 20 | QMUL |
| *N. tabacum* SR1 | *Nicotiana* | tetraploid | 4 | 20 | UCR |
| Synthetic *N. tabacum* QM | synthetic *Nicotiana* | tetraploid | 18 | 87 | both |
| Synthetic *N. tabacum* TH37 | synthetic *Nicotiana* | tetraploid | 1 | 5 | QMUL |
| *N. tomentosiformis* BRNO 4103 | *Tomentosae* | diploid | 5 | 24 | QMUL |
| *N. undulata* PI306637-61C | *Undulatae* | diploid | 5 | 25 | QMUL |
| *N. wigandioides* | *Undulatae* | diploid | 3 | 22 | both |
| TH32 (*N. sylvestris* × *N. otophora*) | synthetic *Sylvestres-Tomentosae* | tetraploid | 5 | 25 | QMUL |

^a^Total number of flowers in analysis, usually five flowers per plant. TW and TH accessions come from the United States *Nicotiana* Germplasm Collection. 9-digit **4750*** accessions come from Radboud University, Nijmegen, The Netherlands. ‘Baldwin’ accessions come from the Baldwin lab (Max Planck Institute, Jena, Germany). CAM accessions come from the Cambridge University Botanic Garden, UK. CPG accessions come from the Chelsea Physic Garden, UK. The 095-55 accession comes from IPK Gatersleben, Germany. 517** accessions were collected in Bolivia; the number refers to the field collection numbers of M. Nee (New York Botanical Garden, USA). *N. ‘rastroensis’* material came from the Smith Lab (University of Colorado, Boulder, Boulder, CO, USA), voucher: Holtsford, *s.n.* and represents an undescribed species.
