# Supplementary material for: Early consequences of allopolyploidy alter floral evolution in Nicotiana (Solanaceae)
Source: BMC Plant Biol. 2019 Apr 27;19:162. doi: 10.1186/s12870-019-1771-5 (PMC6486959; doi:10.1186/s12870-019-1771-5)
Supplement: Supplementary file 8 — Figure S6. Maximum likelihood trees for individual loci. (PPTX 1505 kb) [file 12870_2019_1771_MOESM8_ESM.pptx]

## Slide 1
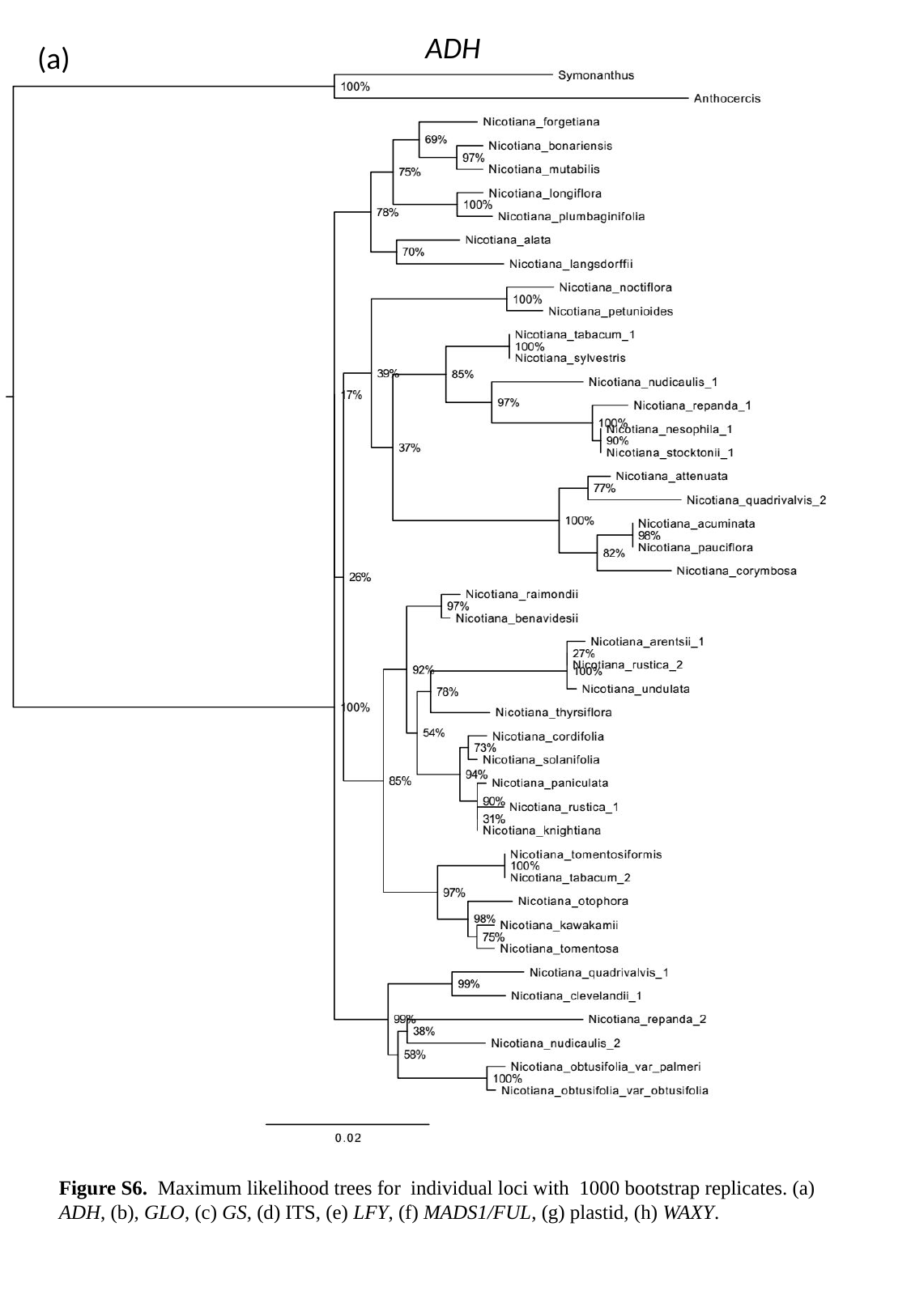

ADH
(a)
Figure S6. Maximum likelihood trees for individual loci with 1000 bootstrap replicates. (a) ADH, (b), GLO, (c) GS, (d) ITS, (e) LFY, (f) MADS1/FUL, (g) plastid, (h) WAXY.

## Slide 2
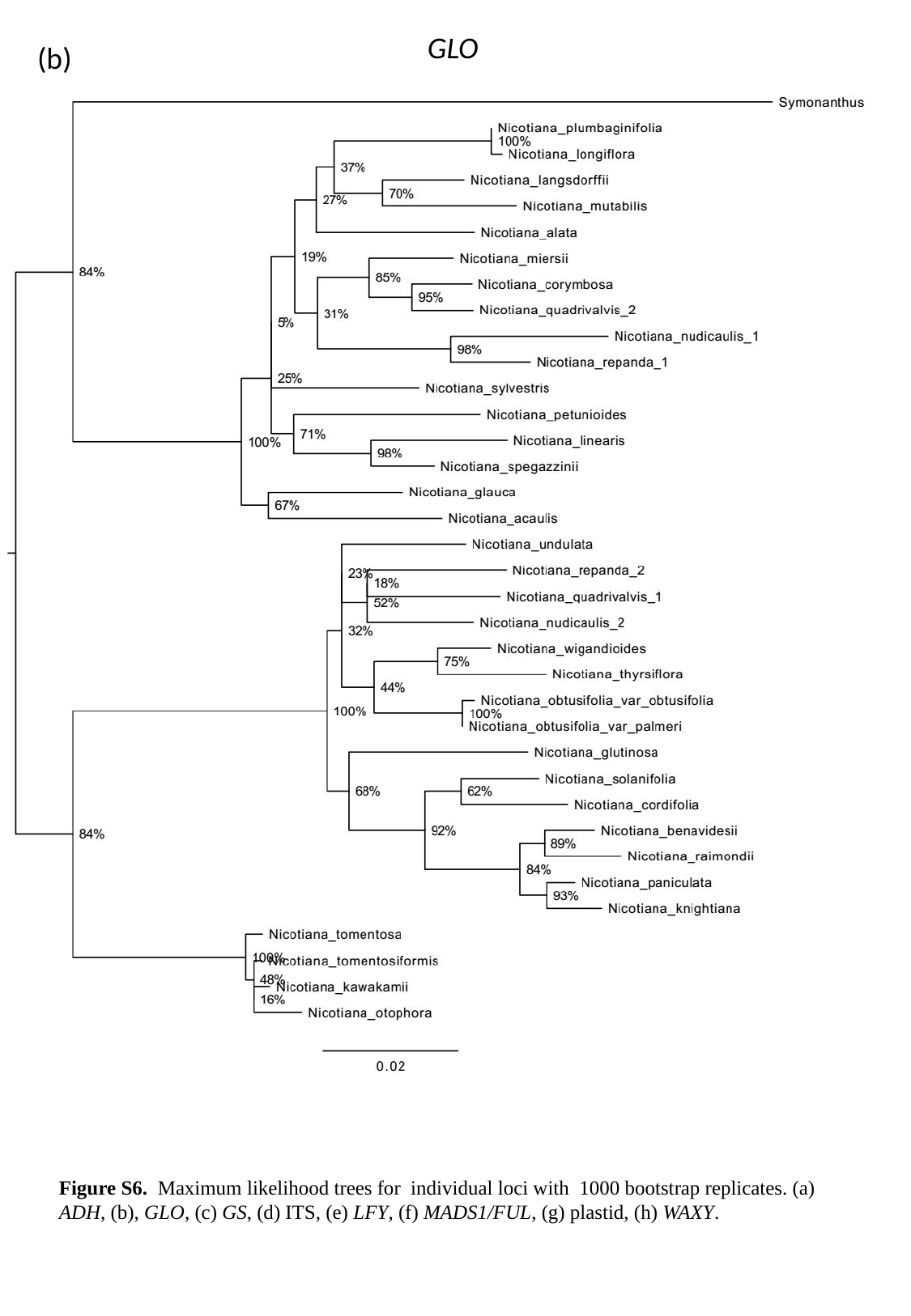

GLO
(b)
Figure S6. Maximum likelihood trees for individual loci with 1000 bootstrap replicates. (a) ADH, (b), GLO, (c) GS, (d) ITS, (e) LFY, (f) MADS1/FUL, (g) plastid, (h) WAXY.

## Slide 3
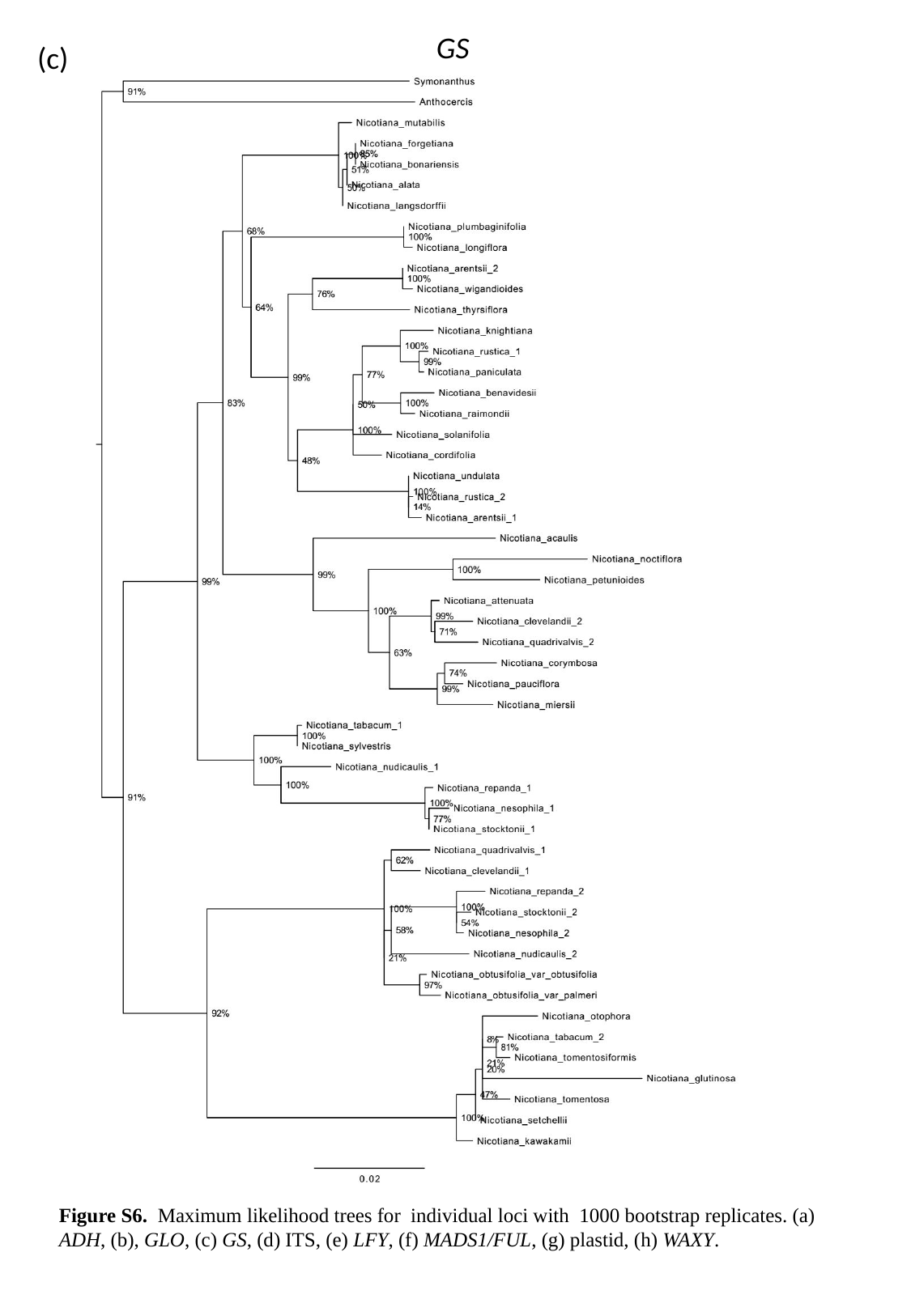

GS
(c)
Figure S6. Maximum likelihood trees for individual loci with 1000 bootstrap replicates. (a) ADH, (b), GLO, (c) GS, (d) ITS, (e) LFY, (f) MADS1/FUL, (g) plastid, (h) WAXY.

## Slide 4
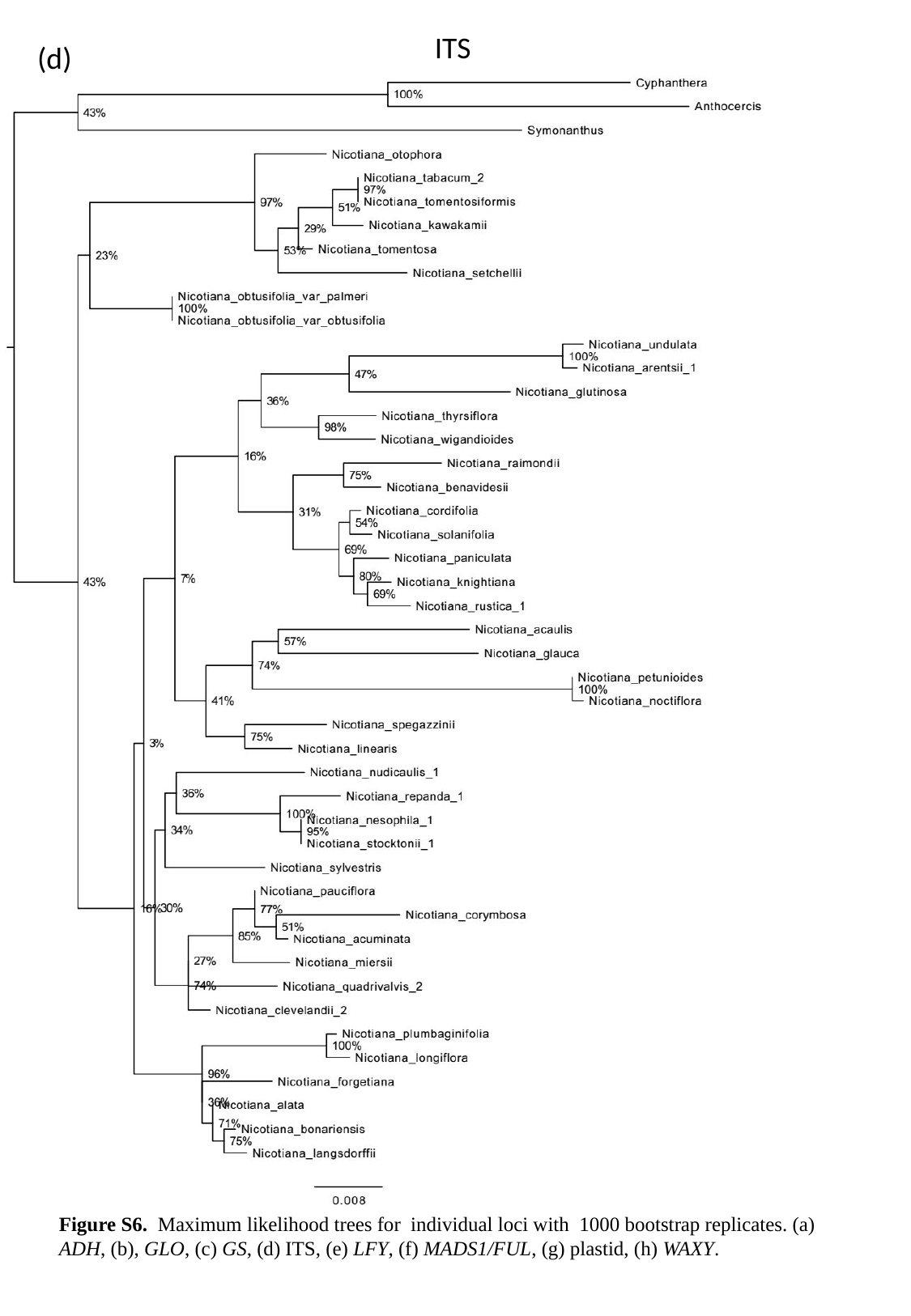

ITS
(d)
Figure S6. Maximum likelihood trees for individual loci with 1000 bootstrap replicates. (a) ADH, (b), GLO, (c) GS, (d) ITS, (e) LFY, (f) MADS1/FUL, (g) plastid, (h) WAXY.

## Slide 5
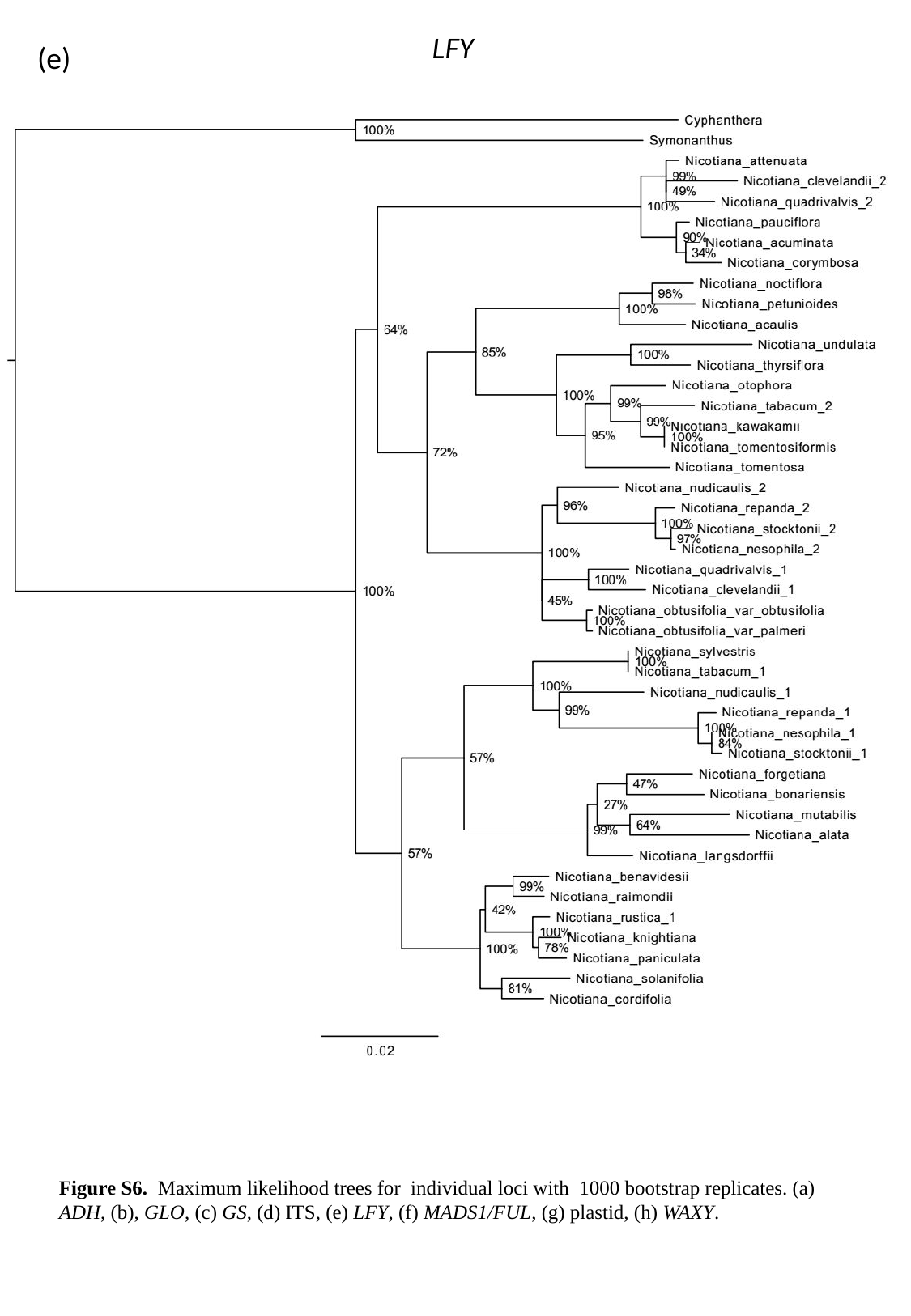

LFY
(e)
Figure S6. Maximum likelihood trees for individual loci with 1000 bootstrap replicates. (a) ADH, (b), GLO, (c) GS, (d) ITS, (e) LFY, (f) MADS1/FUL, (g) plastid, (h) WAXY.

## Slide 6
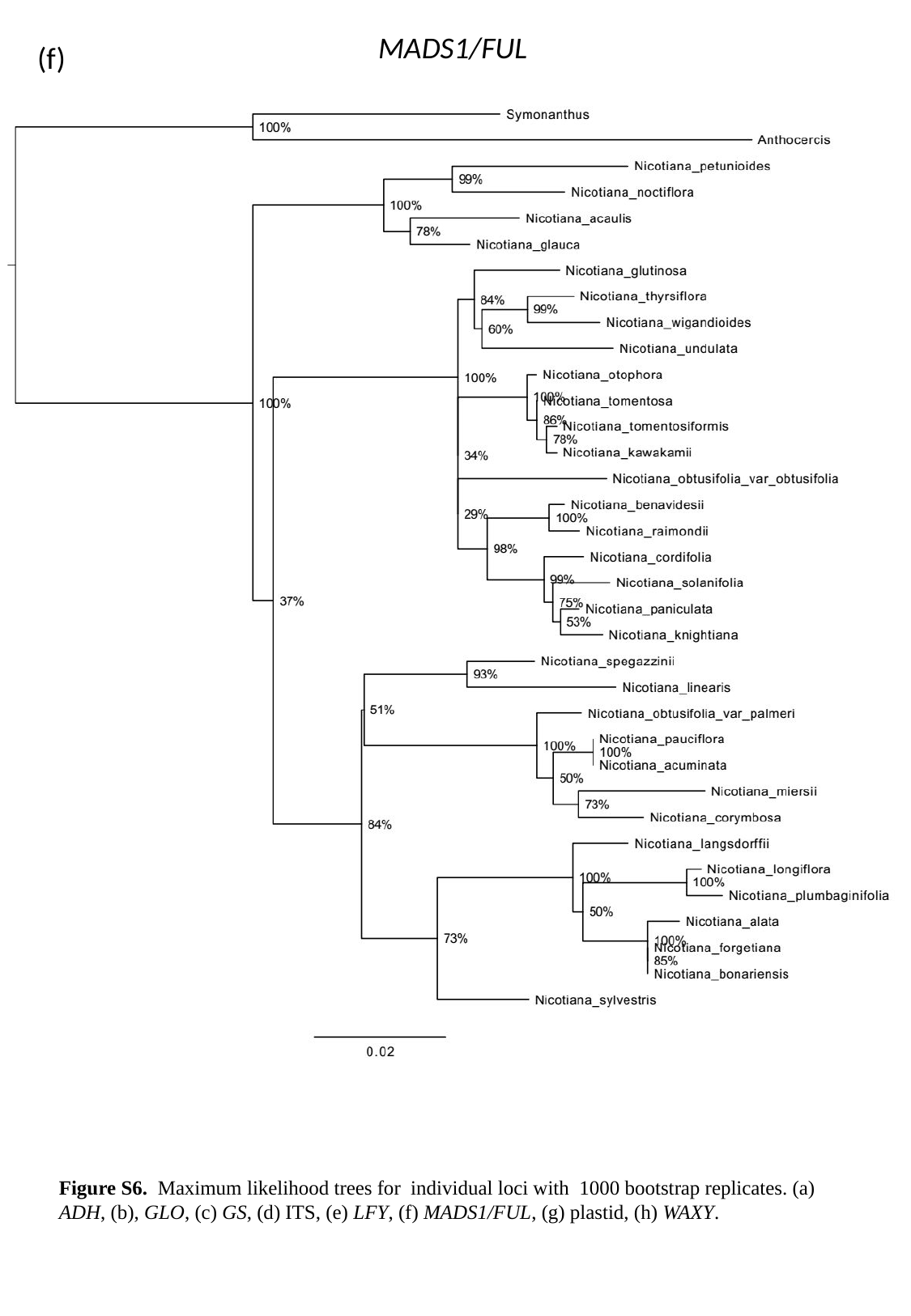

MADS1/FUL
(f)
Figure S6. Maximum likelihood trees for individual loci with 1000 bootstrap replicates. (a) ADH, (b), GLO, (c) GS, (d) ITS, (e) LFY, (f) MADS1/FUL, (g) plastid, (h) WAXY.

## Slide 7
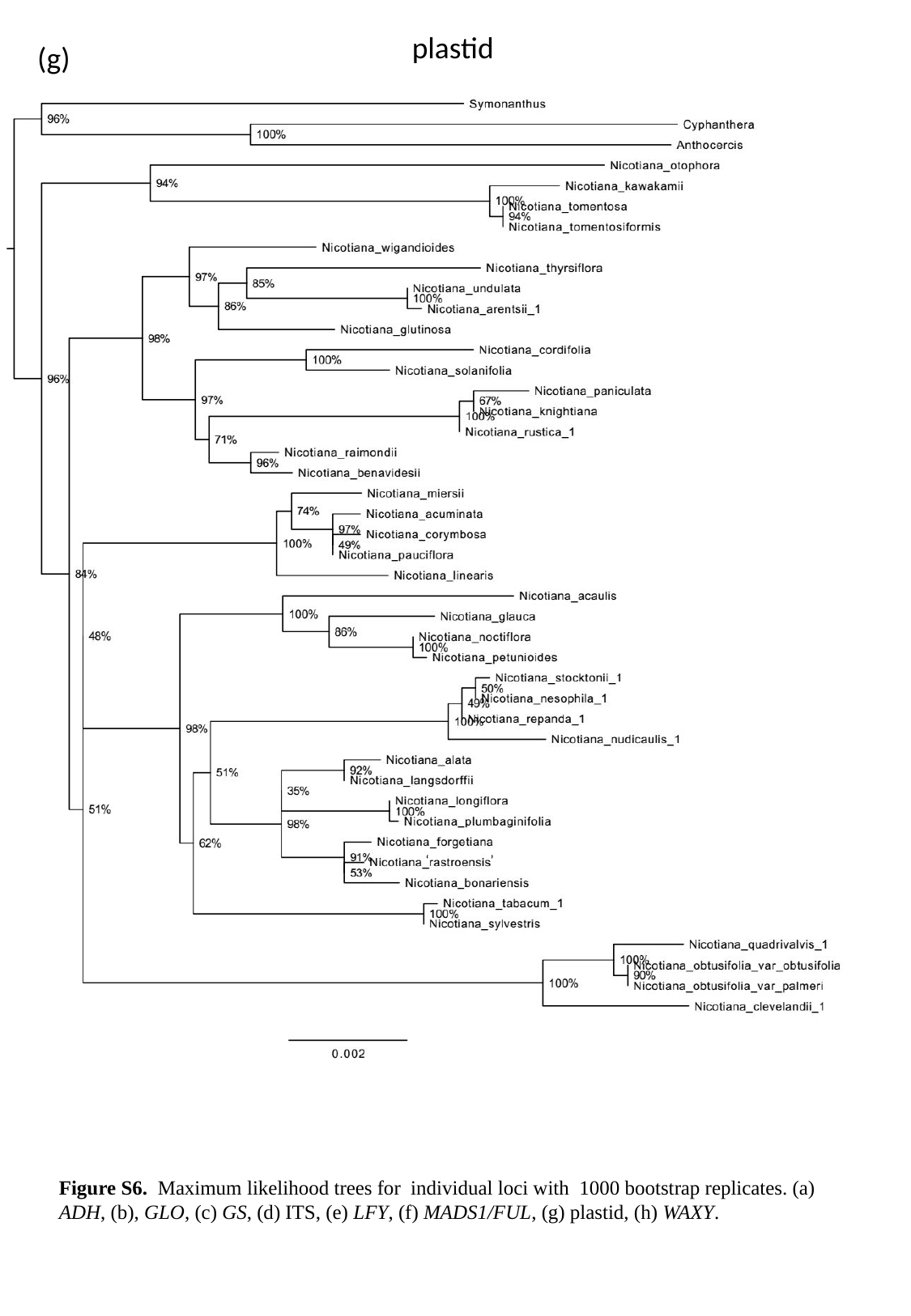

plastid
(g)
‘
’
Figure S6. Maximum likelihood trees for individual loci with 1000 bootstrap replicates. (a) ADH, (b), GLO, (c) GS, (d) ITS, (e) LFY, (f) MADS1/FUL, (g) plastid, (h) WAXY.

## Slide 8
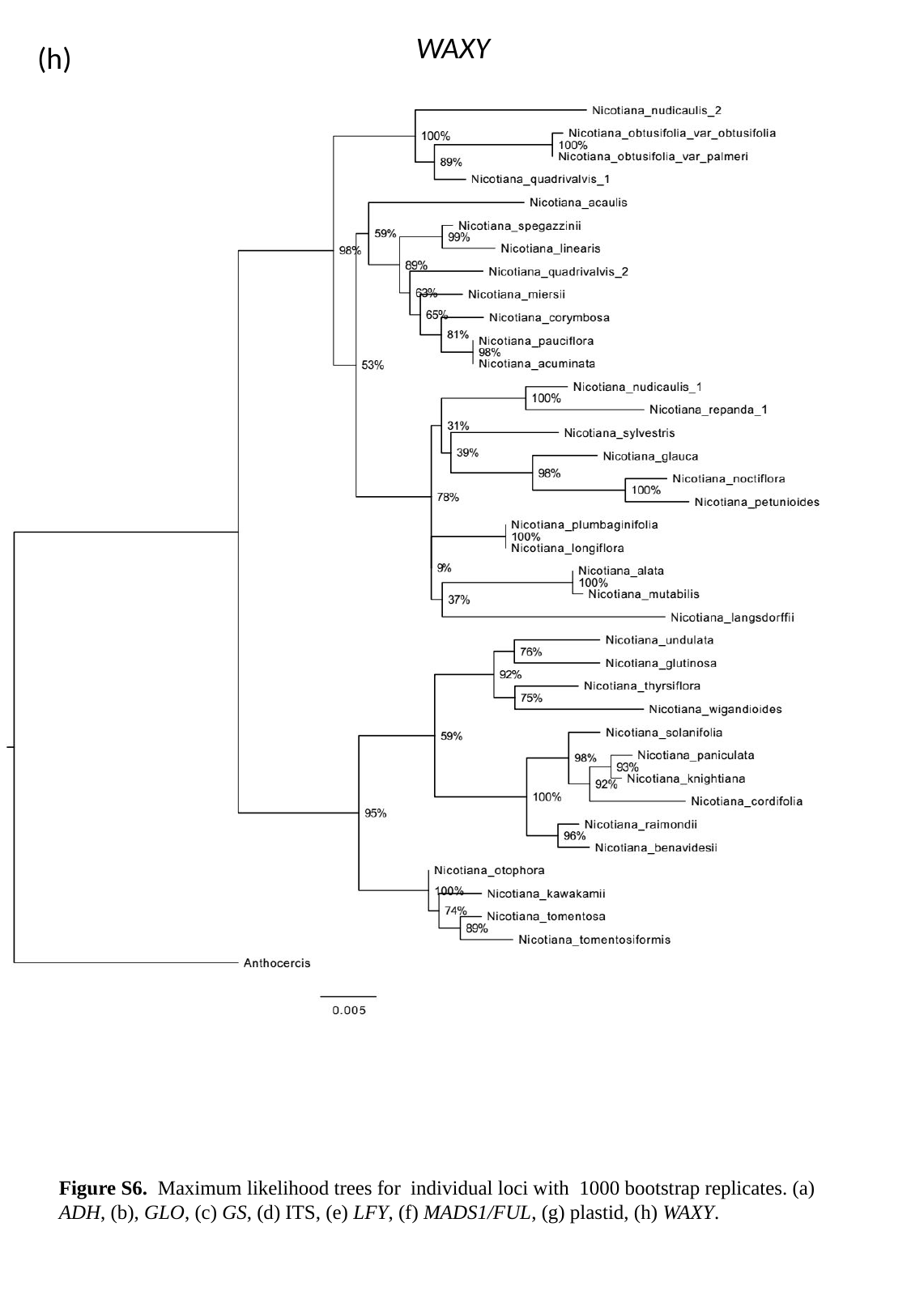

WAXY
(h)
Figure S6. Maximum likelihood trees for individual loci with 1000 bootstrap replicates. (a) ADH, (b), GLO, (c) GS, (d) ITS, (e) LFY, (f) MADS1/FUL, (g) plastid, (h) WAXY.
