# Supplementary material for: Early consequences of allopolyploidy alter floral evolution in Nicotiana (Solanaceae)
Source: BMC Plant Biol. 2019 Apr 27;19:162. doi: 10.1186/s12870-019-1771-5 (PMC6486959; doi:10.1186/s12870-019-1771-5)
Supplement: Supplementary file 9 — Figure S7. Spectra principal components analysis. (PPTX 45 kb) [file 12870_2019_1771_MOESM9_ESM.pptx]

## Slide 1
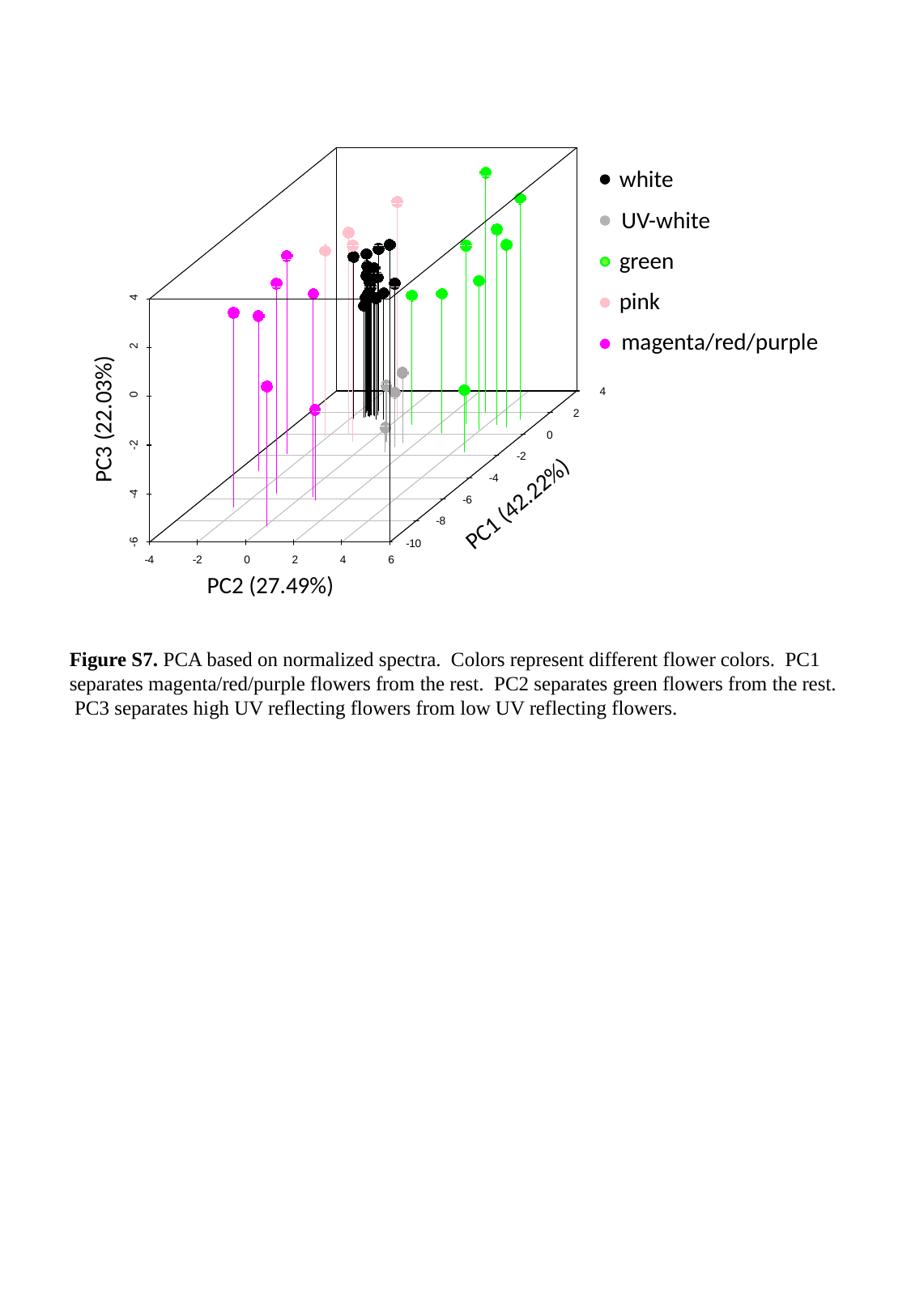

PC3 (22.03%)
PC1 (42.22%)
PC2 (27.49%)
white
UV-white
green
pink
magenta/red/purple
Figure S7. PCA based on normalized spectra. Colors represent different flower colors. PC1 separates magenta/red/purple flowers from the rest. PC2 separates green flowers from the rest. PC3 separates high UV reflecting flowers from low UV reflecting flowers.
